# Short-Wave Infrared InAs Quantum-Dot Light-Emitting Diodes with Tunable Electroluminescence beyond 1.4 μm

**DOI:** 10.1021/acsenergylett.5c03820

**Published:** 2026-02-04

**Authors:** Hossein Roshan, Davide Mazza, Satyaprakash Panda, Francesco de Boni, Luca De Trizio, Liberato Manna, Francesco Di Stasio

**Affiliations:** † Photonic Nanomaterials, 121451 Istituto Italiano di Tecnologia, Via Morego 30, 16163 Genova, Italy; ‡ Università degli Studi di Genova, Via Dodecaneso 31, 16146 Genova, Italy; § Nanochemistry, 121451 Istituto Italiano di Tecnologia, Via Morego 30, 16163 Genova, Italy; ∥ Materials Characterization Facility, 121451 Istituto Italiano di Tecnologia, Via Morego 30, Genova 16163, Italy; ⊥ Chemistry Facility, 121451 Istituto Italiano di Tecnologia, Via Morego 30, 16163 Genova, Italy

## Abstract

Short-wave infrared (SWIR) optoelectronics based on colloidal quantum dots (QDs) have been dominated by heavy-metal chalcogenides for many years (e.g., PbS, HgTe). Here, we present Restriction of Hazardous Substances-compliant InAs/ZnSe core/shell QD light-emitting diodes (LEDs) operating in the SWIR spectral range. The InAs cores are synthesized via a tris­(dimethylamino)­arsine-based continuous-injection method, enabling size control and SWIR spectral tuning. The LEDs employ a hybrid charge-injection stack comprising organic hole-transport layers (poly-TPD/PTAA) and an inorganic ZnMgO electron-transport layer to balance injection into the InAs/ZnSe core/shell QD film. Four different LEDs, with electroluminescence (EL) peaks centered at 1007, 1275, 1300, and 1410 nm, achieve external peak quantum efficiencies of 6.20%, 3.75%, 2.04%, and 1.10%, respectively. This work is the first demonstration of EL from InAs QDs beyond 1100 nm, advancing III–V QDs for SWIR optoelectronic systems in fields such as machine vision and bioimaging.

## Graphical abstract

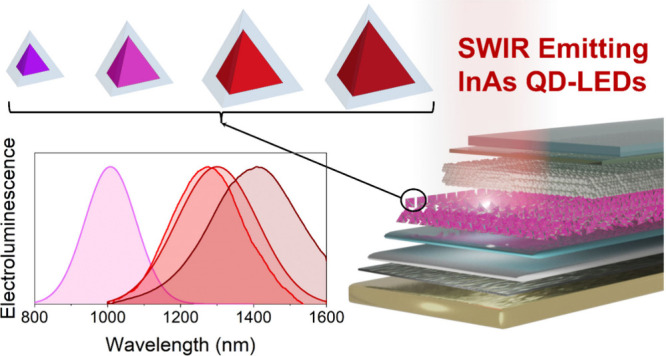

Short-wave infrared (SWIR, 900–1700 nm) light sources underpin emerging optoelectronic platforms in bioimaging,^1^ active imaging,^2^ optical communications,^3^ machine vision, and light detection and ranging (LiDAR).^4^ Currently, SWIR light-emitting diodes (LEDs) are mainly based on single-crystal III–V semiconductors (predominantly In_1–*x*_Ga_*x*_As grown onto InP), whose multistep epitaxial growth and processing make them complex and costly, thus hindering their widespread use in the consumer market.^5^ Moreover, InP serves as the starting substrate onto which In_1–*x*_Ga_*x*_As is epitaxially grown and, for lattice mismatch reasons, the composition of the In_1–*x*_Ga_*x*_As layer is limited to certain stoichiometries, intrinsically restricting the bandgap tunability, thus the emission wavelengths.^6^ Organic semiconductors offer an alternative, presenting large-area, lightweight, and mechanically flexible optoelectronic devices; however, their efficiency drops sharply for emission beyond 800 nm, limiting their suitability for SWIR applications.^7,8^ Additional SWIR light sources are colloidal quantum dots (QDs), as they can be synthesized at a price considerably lower than that required for epitaxial crystals. Furthermore, they are not grown on a substrate; thus, they do not require lattice matching nor are they bound to composition constraints. As a result, they can be readily integrated with well-established and inexpensive complementary metal-oxide-semiconductor (CMOS) technologies.^9^ Despite the commercial success of visible QD displays, SWIR QD light sources are not yet consumer-grade, given the difficulty in achieving high efficiency for emissions beyond 1100 nm in combination with compliance with the European Restriction of Hazardous Substances (RoHS) directive. In fact, state-of-the-art SWIR QD-LEDs are dominated by toxic lead and mercury chalcogenide QDs: PbS QDs in a blended binary-matrix architecture have reached an external quantum efficiency (EQE) of 7.9% at 1400 nm and 11.8% at 1550 nm.^10,11^ Mercury-alloyed nanoplatelets (CdHgSe/ZnCdS) have delivered electroluminescence (EL) from 1300 to 1550 nm with an EQE up to 7.5%,^12,13^ and HgTe QD-LEDs span the 1100–1700 nm range, albeit at modest efficiencies (≈0.7–2.2%).^2,14^ Among heavy-metal-free QDs, which comprise silver-chalcogenides,^15–19^ Cu–In-chalcogenides,^20–23^ and III–V semiconductors,^24^ InAs QDs are uniquely positioned for SWIR emission: thanks to their narrow bandgap in bulk (0.35 eV)^25^ and a large exciton Bohr radius (≈45 nm),^26^ their absorption and emission can be tuned from 650 nm up to 3500 nm (as demonstrated in epitaxial InAs LEDs).^27,28^ On the other hand, progress on colloidal InAs-based QDs has been constrained by reactivity limits of available As precursors, leading to QDs with low photoluminescence quantum yield (PLQY), size-dispersion challenges, and EL limited to wavelengths below ≈1006 nm^29^ (although, Tessler et al. in 2002 demonstrated emission from InAs/ZnSe QDs at 1270 nm via energy transfer from a conjugated polymer in organic-LEDs).^30^ From a synthesis standpoint, colloidal InAs QDs used in LEDs are made with either tris­(trimethylsilyl)­arsine (TMS-As) or tris­(dimethylamino)­arsine (amino-As) precursors. The former (TMS-As) enables the synthesis of InAs QDs with narrow optical features and high optical quality.^31–33^ For example, Wijaya et al.^34^ reported the synthesis of TMS-As-based In­(Zn)­As/multishell QDs emitting at 850 nm with a PLQY of 75%. These QDs enabled the fabrication of RoHS-compliant LEDs exhibiting a peak EQE of 4.6% at 857 nm. Through subsequent optimization of both core and shell synthesis parameters, as well as device architecture, the same group achieved In­(Zn)­As/multishell QDs with emission red-shifted to 985 nm and a PLQY of 73%, leading to LEDs with a peak EQE of 13.3% at 1006 nm.^29^ Similarly, Li et al.^35^ reported InAs/InP/ZnSe/ZnS QDs with PLQY approaching 100% at 900 nm, with the corresponding LEDs featuring an EQE of 20.5% at 905 nm. Despite the good results and performance of InAs QDs produced via the TMS-As route, such a precursor is expensive, pyrophoric, and difficult to source. This has driven research toward cheaper and safer alternative As precursors, among which amino-As currently represents the most promising option.^36,37^ Indeed, it is now possible to synthesize amino-As-based InAs/ZnSe core/shell QDs with high PLQY.^38^ Employing amino-As-based InAs/ZnSe QDs with ∼40% PLQY, our group demonstrated QD-LEDs with EL at 947 nm and an EQE of 5.5%.^39^ After improving the ZnSe shelling protocol, we were able to prepare InAs/ZnSe QDs with a thick shell (∼7 monolayers) and PLQY around 70% at 900 nm.^40^ The resulting LEDs achieved an EQE of 13.3% at 896 nm, outperforming PbS QD-LEDs.^41^ Importantly, all these results were obtained by employing relatively small InAs QDs, restricting the observed EL to wavelengths below 1100 nm. In fact, until recently, no synthesis routes to large InAs QDs with efficient PL beyond 1000 nm had been reported using either amino-As or TMS-As precursors, thus creating a key roadblock to further development of InAs QD-LEDs.^42–45^ To start filling this gap, in this work, we fabricated LEDs by employing our recently reported large, SWIR-emissive amino-As-based InAs/ZnSe QDs.^46^ The QDs feature a PLQY as high as 45% at 966 nm, with the emission peak tunable up to 1430 nm, where a PLQY of 23% is achieved. We fabricated LEDs based on such QDs by employing an optimized ZnMgO electron transport layer (ETL) and a double organic hole transport layer (HTL) to balance injections and confine recombination in the active material. In this architecture, we obtained a 6.20% EQE at 1007 nm and a 1.10% EQE at 1410 nm. To the best of our knowledge, this is the first demonstration of EL > 1100 nm from RoHS-compliant InAs QDs.

The InAs/ZnSe core/shell QDs used here were synthesized using our recently published seeded growth approach.^46^ Briefly, amino-As-based InAs QDs with an excitonic absorption peak at ∼950 nm were first synthesized employing ZnCl_2_ as an additive and a novel reducing agent, namely, trioctylamine-alane (TOA-AlH_3_), and subsequently grown to larger sizes by adding InCl_3_, followed by the continuous co-injection of amino-As and TOA-AlH_3_. The schematic of the seeded growth synthesis is shown in Figure 1a. After reaching the desired size, the InAs QDs were coated with 6–8 monolayers of ZnSe grown *in situ* at 300 °C, employing ZnCl_2_ dissolved in oleylamine and selenium powder in trioctylphosphine. XRD analysis before (Figure S1) and after shelling (Figure 1b) confirmed the successful overgrowth of a zincblende ZnSe shell onto InAs cores. In total, we prepared four InAs/ZnSe QDs with different sizes, namely, 10.4 ± 1.5 nm (QD1) starting from 4.6 ± 0.4 nm InAs cores, 12.7 ± 1.6 nm (QD2) from 5.2 ± 0.5 nm InAs cores, 12.9 ± 1.4 nm (QD3) from 5.7 ± 0.5 nm InAs cores, and 13.0 ± 1.8 nm (QD4) from 6.3 ± 0.7 nm InAs cores (Figure 1c–f and Figure S2). The size distribution histograms of core/shell QDs are shown in Figure S3. The ZnSe shell thickness (reported as ∼6–8 monolayers) was estimated by combining the QD size increase extracted from TEM with elemental quantification by Inductively Coupled Plasma Optical Emission Spectroscopy (ICP-OES) and interpreting the data using the core/shell structural model previously established for this tetrahedral InAs/ZnSe system.^40^

**Figure 1:**
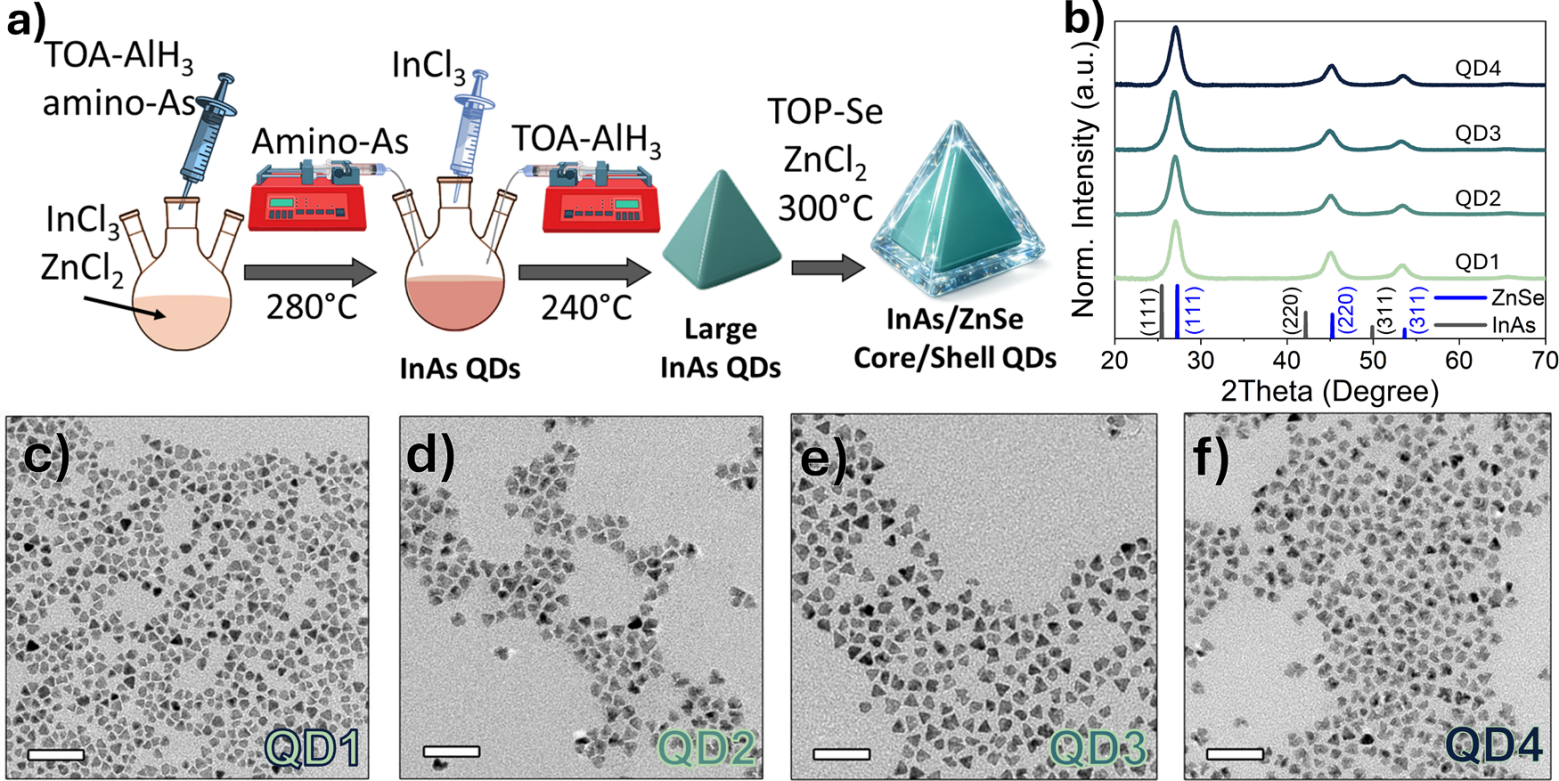
(a) Schematic of the seeded growth approach employed to obtain InAs/ZnSe core/shell QDs. (b) XRD pattern of the InAs/ZnSe core/shell QDs. QD1 to QD4 are ordered from smallest to largest core based on TEM-derived mean particle size. Transmission electron microscopy (TEM) images of InAs/ZnSe core/shell QDs: (c) QD1, (d) QD2, (e) QD3, and (f) QD4. Scale bar in TEM images is 50 nm.

Figure 2 summarizes the steady-state optical properties and electronic band structures of the QDs. Figure 2a–d presents the optical absorption spectra of the InAs cores and InAs/ZnSe core/shell QDs, along with the PL spectra of the respective core/shell QDs. The PL spectra of the InAs/ZnSe QDs in solution exhibit emission peaks at 966 nm (QD1, full-width at half-maximum, fwhm = 198 meV, and PLQY = 45 ± 4%), 1267 nm (QD2, fwhm = 177 meV, PLQY = 35 ± 4%), 1370 nm (QD3, fwhm = 177 meV, PLQY = 30 ± 3%), and 1410 nm (QD4, fwhm = 165 meV, PLQY = 17 ± 2%). The obtained line widths are also in line with other SWIR-emissive colloidal QDs: Ag_2_S QDs with emission around 1250 nm have fwhm ≈200 nm,^47^ CdHgSe-based QDs have fwhm of 118–123 meV depending on emission wavelength,^12^ while colloidal InSb QDs can exhibit PL at 1507 nm with fwhm around 188 meV .^47^ Time-resolved photoluminescence (TRPL) of all QDs is reported in Figure S4 (see also Table S1). Figure 2e shows the PLQY values for QD1–QD4. As the emission shifts to longer wavelength (from QD1 to QD4), the PLQY decreases steadily from 45 ± 5% to 17 ± 2%. Noticeably, the PLQY of all samples in solution is below 50%, indicating that further improvement in their synthesis is needed to reach higher values, including shell materials with better lattice matching than ZnSe and *ad-hoc* surface passivation of InAs QDs prior to shelling. Table 1 summarizes the optical characteristics of the core/shell InAs/ZnSe QDs where a further drop in the PLQY is observed when moving from solution to film. Such a PLQY drop is commonly observed, and it can be assigned to a variety of phenomena ranging from energy transfer between neighboring QDs to ligand rearrangement and desorption. Importantly, the PLQY drop is quite pronounced, suggesting that the interfacial/surface defects on the QDs are very effective at quenching the PL when in a closely packed solid such as the films here studied.

**Figure 2:**
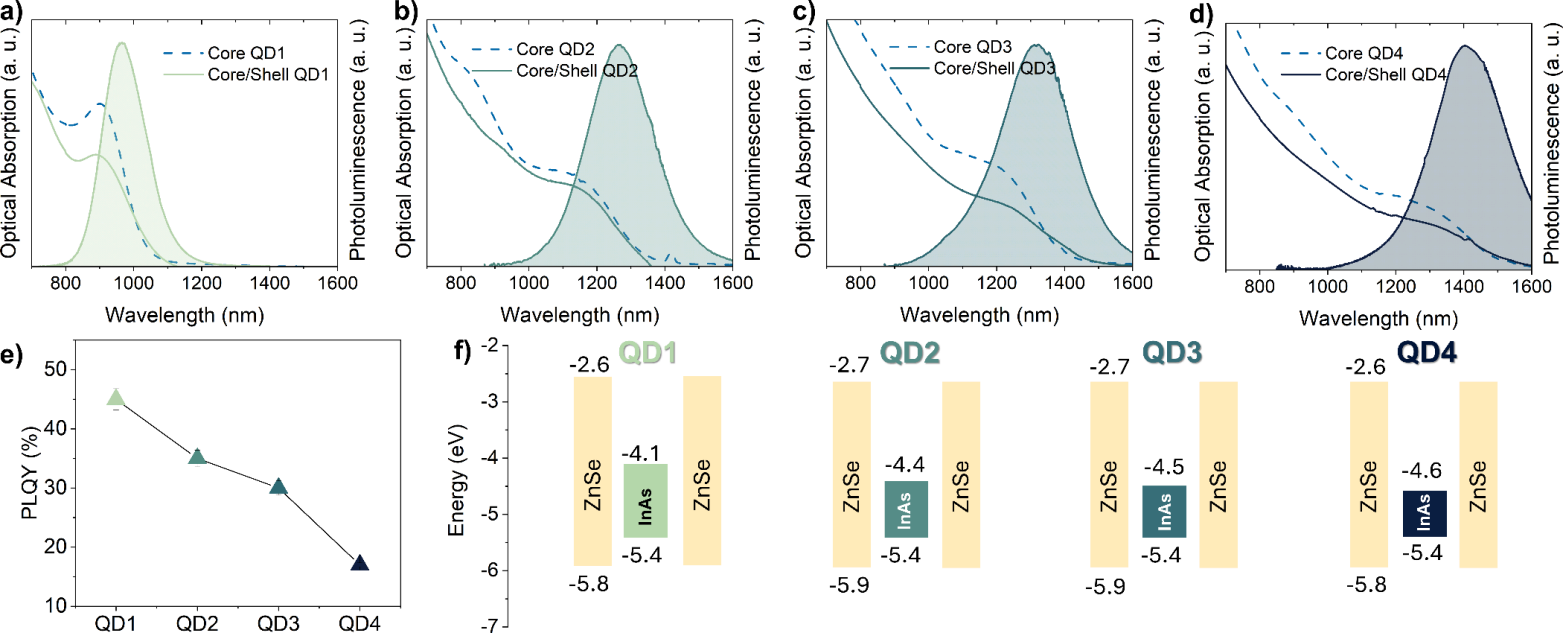
Photoluminescence of InAs/ZnSe core/shell QDs in solution, absorption of the respective core QDs (dashed lines), and absorption of core/shell QDs: (a) QD1, (b) QD2, (c) QD3, and (d) QD4. (e) Evolution of the PLQY across the sample set. (f) Flat band energy diagram of core/shell InAs/ZnSe QDs. The valence band maxima with respect to vacuum were determined by UPS, while the conduction band minima with respect to vacuum were derived from the optical bandgap obtained by Tauc plot analysis for InAs cores and an assumed bandgap of 3.2 eV for 6–8 monolayer-thick ZnSe shells. The flat band values have been rounded up to the last significant figure, considering an error of ±0.1 eV arising from the UPS measurements and analysis.

**Table 1:** Summary of the Photoluminescence Characteristics of the QDs Used in This Study

Sample	PL peak (nm)	PLQY (%)	fwhm (meV)	PLQY in film (%)
QD1	966	45 ± 5	198	25 ± 3
QD2	1267	35 ± 4	177	12 ± 1
QD3	1370	30 ± 3	177	10 ± 1
QD4	1410	17 ± 2	165	9 ± 1

The core-only InAs and core/shell InAs/ZnSe QDs were analyzed by X-ray photoelectron spectroscopy (XPS) and ultraviolet photoelectron spectroscopy (UPS, Figures S5–S8), which are in agreement with our previous works.^38,40^ UPS measurements revealed that the valence band maximum (VBM) of core-only InAs QDs is around −5.4 ± 0.1 eV for all four samples (vacuum-referenced, ± 0.1 eV is the estimated error derived from the UPS analysis) and shifted to −5.8 ± 0.1 eV upon ZnSe shelling, indicating an energy step around 0.4 eV between core and shell VBM. Assuming the UPS signal is dominated by the outer ZnSe shell layers and that the 6–8 monolayer shell bandgap is around 3.2 eV,^49^ Figure 2f plots the resulting band alignment by combining all VBM values with the core-only InAs QD bandgaps, which were extracted from their corresponding Tauc plots (i.e., 1.24 eV for the core of QD1, 0.96 eV for QD2, 0.92 eV for QD3, and 0.85 eV for QD4, see Figure S9). The resulting energy offset between cores and shells indicates a type I heterojunction in all QDs.

Upon completion of the characterization, we then employed our QDs as active materials in LEDs. Initially, we employed an inverted QD-LED stack previously optimized for InAs/ZnSe QDs emitting at 900 nm: ITO (indium tin oxide)/ZnO/PMMA/QDs/Poly-TPD/MoO_*x*_/Al (Figure S10a).^41^ In this architecture, a thin PMMA layer atop ZnO moderates electron injection into the active layer and increases the ZnO-QD separation, thus reducing quenching from image charge effects, while Poly-TPD (poly­[*N*,*N*′-bis­(4-butylphenyl)-*N*,*N*′-bisphenylbenzidine]) promotes hole injection. In the present work, we systematically varied the thicknesses of the transport layers to improve charge balance in the devices; however, the resulting devices exhibited an EQE ≤ 0.5% (Figure S10b) for all QDs. The persistently high current density (both below and above the turn-on voltage) is the main cause of the low EQE. To address this issue, we had to modify the starting architecture by varying the composition of both the hole-transport (HTL) and electron-transport layers (ETL).

Figure 3a illustrates the final architecture, and Figure 3b displays the energy levels and band alignment corresponding to the various layers. The stack starts from glass/ITO, Zn_0.9_Mg_0.1_O as ETL, InAs/ZnSe core/shell QD film as the active layer, a double-HTL of poly-TPD followed by PTAA (poly­[bis­(4-phenyl)­(2,4,6-trimethylphenyl)­amine]), a thin MoO_*x*_ interlayer that raises the effective anode work function, inducing favorable band bending and promoting hole injection,^50^ and finally an evaporated gold top contact. We replaced ZnO with Zn_1–*x*_Mg_*x*_O (0.05 ≤ *x* ≤ 0.40) to improve the energy alignment of the ETL layer with the QDs, as Zn_1–*x*_Mg_*x*_O exhibits an approximately linear bandgap increase with increasing Mg content, shifting the CBM closer to vacuum level.^51^ With increasing Mg content, the electron mobility decreases,^52^ while also donor and trap levels become deeper, and the electrical conductivity consequently decreases.^53^ By modulating the Mg content in ZnO, we raised the CBM of the ETL from −4.2 (pure ZnO)^41^ to −3.7 eV (final composition: Zn_0.9_Mg_0.1_O, Figure S11), thus reducing the electron-injection barrier at the ETL/QD interface.^54^ The measured offsets place the Zn_0.9_Mg_0.1_O CBM above the InAs QDs CBM; thus electrons encounter only a modest barrier introduced by the 6–8 monolayers of ZnSe, enabling efficient electron injection. In addition, Zn_0.9_Mg_0.1_O injects fewer electrons, as it is less conductive than ZnO (≈10^–3^ for ZnO to ≈10^–5^ cm^2^ V^–1^ s^–1^ for Zn_1–*x*_Mg_*x*_O),^55^ resulting in a better charge balance in the active layer and performance enhancement. Regarding HTLs, the HOMO (highest occupied molecular orbital) of Poly-TPD is reported to be around −5.3 eV,^56,57^ well aligned with the VBM of core-only InAs QDs (see Figure 2f). Also, Poly-TPD exhibits a hole mobility of 10^–5^–10^–4^ cm^2^ V^–1^ s^–1^.^58,59^ PTAA, with a reported HOMO level around −5.2 eV,^60–62^ provides improved hole mobility compared to poly-TPD (≈10^–3^ cm^2^ V^–1^ s^–1^),^63,64^ promoting hole transport to the narrow-gap QD layer, while the MoO_*x*_ interlayer reduces the energy barrier between the anode and PTAA. Au was selected as the anode because it forms a stable, oxidation-resistant interface with MoO_*x*_. In fact, we avoided aluminum to prevent interfacial oxidation and degradation.^65^ The scanning electron microscopy (SEM) cross-sectional image is shown in Figure 3c. From top to bottom (thickness values in parentheses): ITO (150 nm)/ZnMgO (78 nm)/InAs/ZnSe (59 nm)/poly-TPD (14 nm)/PTAA (16 nm)/MoO_*x*_ (15 nm)/Au (80 nm). The LED radiance and current density versus voltage (*J–V–R*) curves indicate that the 59 nm QD film is thick enough to generate sufficient light and yield a continuous, pinhole-free layer; yet, it is thin enough to avoid excessive series resistance that would result in high turn-on voltage. With the stack and band alignment established, Figure 3d compares the *J*–*V*–*R* characteristics of the four QD-LEDs. The peak radiance decreases in LEDs with longer emission wavelengths: QD1 and QD2 reach ≈2.3 W sr^–1^ m^–2^, QD3 1.9 W sr^–1^ m^–2^, and QD4 1.0 W sr^–1^ m^–2^. The QD1 LED turns on at the lowest current density and climbs sharply, while the QD4 LED needs noticeably higher current to start emitting, and the final radiance is limited to a lower absolute value. The other informative feature of the *J*–*V*–*R* curves is the turn-on voltage. In all devices, the turn-on sits slightly above the optical bandgap, an offset that reflects injection barriers and series resistance introduced by the double-HTL and the ZnMgO. The turn-on voltage follows the emitter bandgap: QD1 LED, with the widest gap, turns on at the highest applied bias (1.6 V), while the QD4 LED turns on at the lowest one (1.1 V). The final radiance ceiling still follows the PLQY of the respective QD sample. Figure 3e plots EQE versus current density; peak EQEs are 6.20% at 0.24 mA cm^–2^ for QD1 LED (radiance ≈0.059 W sr^–1^ m^–2^), 3.75% at 0.20 mA cm^–2^ for QD2 (≈0.023 W sr^–1^ m^–2^), 2.04% at 0.20 mA cm^–2^ for QD3 (≈0.012 W sr^–1^ m^–2^), and 1.10% at 0.21 mA cm^–2^ for QD4 (≈0.006 W sr^–1^ m^–2^). The corresponding EQE–voltage characteristics are listed in Figure S12. Two systematic behaviors can be concluded from EQE curves and peak EQE points. First, given that the same LED architecture has been employed for all four QDs, the current density range that yields the EQE maxima occurs within a narrow range (0.20–0.24 mA cm^–2^) for all four QD-LEDs. Therefore, with optical outcoupling and charge balance held approximately constant by design, the difference in peak EQE primarily reflects differences in the emissive layer, i.e., the PLQY of the four InAs/ZnSe QD films (see Table S1).^66^ Second, although current-density at peak EQE is nearly invariant, the bias voltage required to reach the EQE maximum shifts modestly among devices, which we attribute to small variations in injection barriers and built-in potential arising from the different QD bandgaps (and hence band alignments) as emission is tuned from 1007 to 1410 nm. At high current densities, all devices are reduced to nearly 50% of the peak EQE value, known as EQE roll-off, which we can attribute to effects such as field-assisted exciton dissociation, Auger recombination at high carrier density, and Joule heating.^67^ The roll-off is more pronounced for QDs emitting at longer wavelengths, consistent with a larger contribution from trap-assisted nonradiative recombination (suggested by the decreasing PLQY from QD1 to QD4). Figure S13 presents statistical EQE distributions for LEDs made with different QDs (QD1–QD4). The boxplots (dots: individual pixels; box: median and interquartile range; whiskers: range) confirm the systematic drop in EQE in large QDs, consistent with the trend discussed in the main text. Figure 3f presents the EL spectra with peaks centered at 1007, 1275, 1300, and 1410 nm. EL peak positions closely track the PL of QDs, indicating that radiative recombination occurs within the InAs cores and electric-field-induced Stark shifts are not observed within the operation range. Table 2 summarizes the LED performance indicators. Finally, Figure S14 shows the operational stability of the devices under fixed current density. The operational stability measurements reveal that the decay of normalized EL intensity under constant-current operation is faster in devices based on larger InAs QDs. In detail, the *T*_50_ (time required for the EL to reach 50% of its initial value) of LEDs based on QD1, QD2, QD3, and QD4 were 31, 28, 21, and 19 min, respectively.

**Figure 3:**
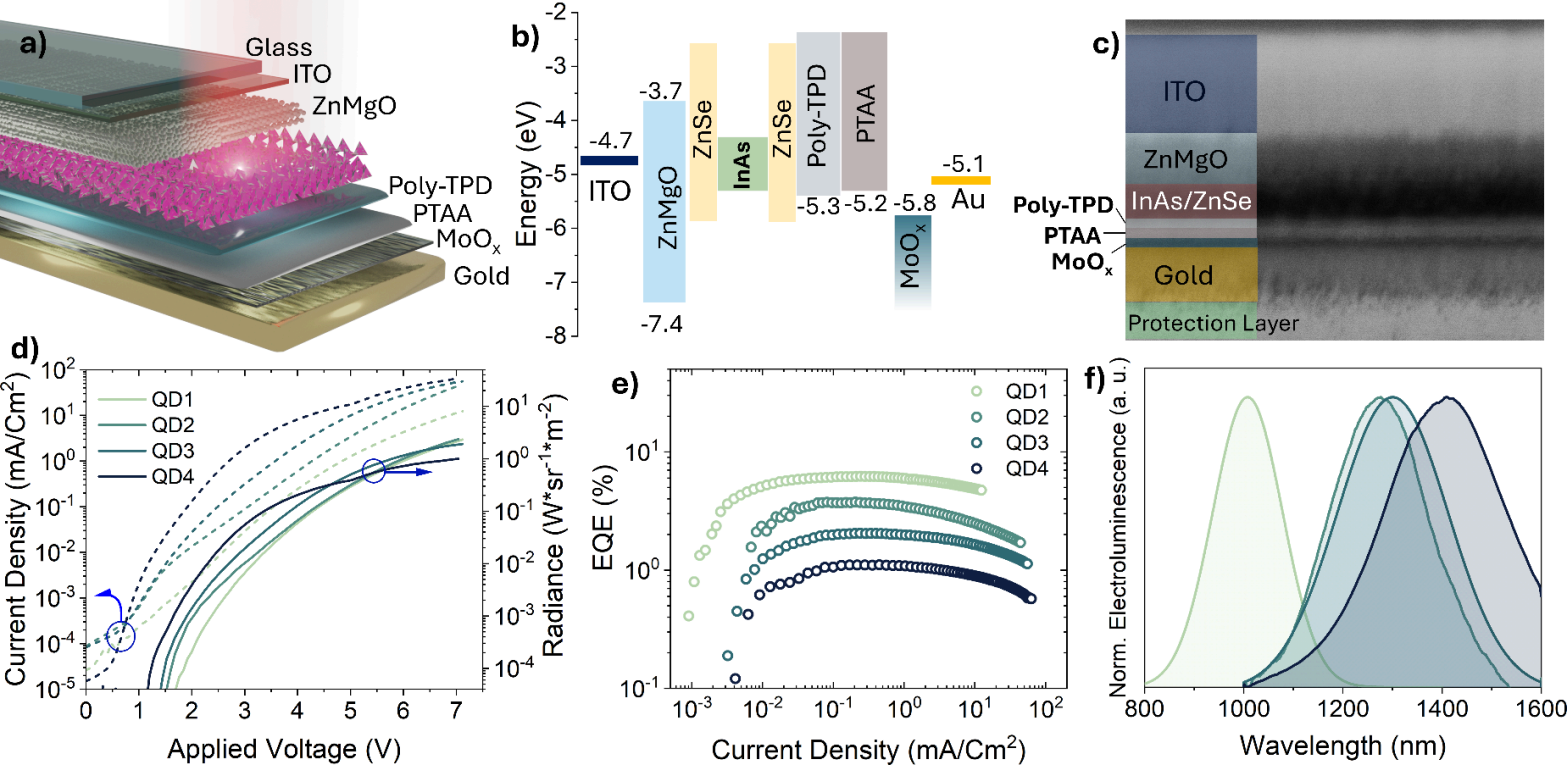
(a) Visualization of the QD-LED structure. (b) Flat band energy diagram of QD-LEDs fabricated in this study. (c) SEM cross-sectional image of a full-stack QD-LED. (d) Current–voltage–radiance curves of all four QD-LEDs. (e) EQE curves versus current density of all devices. (f) EL spectrum of QD-LEDs.

**Table 2:** Summary of QD-LED Performance Metrics in This Study

Device (QD)	EL peak (nm)	EQE peak (%)	*J* @ EQE peak (mA cm^**–**2^)	Radiance @ EQE peak (W sr^**‑**1^ m^**–**2^)	Max radiance (W sr^**‑**1^ m^**–**2^)
QD1	1007	6.20	0.24	0.059	2.3
QD2	1275	3.75	0.20	0.023	2.3
QD3	1300	2.04	0.20	0.012	1.9
QD4	1410	1.10	0.21	0.006	1.0

In conclusion, we demonstrated RoHS-compliant amino-As-based InAs/ZnSe QD-LEDs with EL tunable across 1007–1410 nm and a peak EQE of 6.2%, comparable to Pb-based QD-LEDs. Notably, this is the first efficient InAs QD-LED with emission beyond 1100 nm, establishing a sustainable, solution-processed platform that spans the 1000–1400 nm SWIR window. Benchmarking this work against state-of-the-art SWIR QD-LEDs of different compositions (Table S2) highlights InAs QD-LEDs competitive efficiency in the 1000–1100 nm range and a promising result beyond 1100 nm up to 1410 nm. Further gains are expected from improved surface passivation, for example, via the overgrowth of an external ZnS shell of proper thickness and core/shell interface engineering, along with refined charge-transport layers to boost performance and radiance. Collectively, these results position InAs/ZnSe QD-LEDs as a promising route to scalable, heavy-metal-free SWIR emitters for communication, sensing, and imaging.

## Supplementary Material



## Notes

Experimental section, TEM images of core InAs QDs, XRD patterns of core InAs QDs, XPS/UPS of core InAs versus InAs/ZnSe core/shell QDs (QD1–QD4), Tauc plot of core InAs QDs, initial LED architecture and corresponding EQE, UPS/Tauc of Zn_0.9_Mg_0.1_O, EQE–voltage curves, EQE statistics across multiple pixels, comparison table benchmarking SWIR QD-LEDs (PDF)

## References

[1] Wang F., Ren F., Ma Z., Qu L., Gourgues R., Xu C., Baghdasaryan A., Li J., Zadeh I. E., Los J. W. N., Fognini A., Qin-Dregely J., Dai H. (2022). In Vivo Non-Invasive
Confocal Fluorescence Imaging beyond 1,700 Nm Using Superconducting
Nanowire Single-Photon Detectors. Nat. Nanotechnol..

[2] Bossavit E., Qu J., Abadie C., Dabard C., Dang T., Izquierdo E., Khalili A., Gréboval C., Chu A., Pierini S., Cavallo M., Prado Y., Parahyba V., Xu X. Z., Decamps-Mandine A., Silly M., Ithurria S., Lhuillier E. (2022). Optimized
Infrared LED and Its Use in an All-HgTe Nanocrystal-Based Active Imaging
Setup. Adv. Opt. Mater..

[3] Bao C., Xu W., Yang J., Bai S., Teng P., Yang Y., Wang J., Zhao N., Zhang W., Huang W., Gao F. (2020). Bidirectional Optical
Signal Transmission between Two Identical Devices
Using Perovskite Diodes. Nat. Electron..

[4] Chen W., Wang Y., Chen H., Liu Y. (2022). EIL-SLAM: Depth-enhanced
Edge-based infrared-LiDAR SLAM. J. Field Robot..

[5] Park S., Nguyen P. D., Kim Y., Jeon J., McCartney M. R., Smith D. J., Kim M., Kim D., Chun B. S., Lee S. J. (2024). Metamorphic InGaAs/InAsPSb Quantum
Well Light Emitting
Diodes for Operation in the Short-Wave Infrared Region. Adv. Funct. Mater..

[6] Ning C.-Z., Dou L., Yang P. (2017). Bandgap Engineering in Semiconductor Alloy Nanomaterials
with Widely Tunable Compositions. Nat. Rev.
Mater..

[7] Ahadzadeh S., Brebels S., Maes W., Deferme W. (2025). Strategies for Advancing
Near-Infrared Organic Light-Emitting Diodes: Innovations in Luminescent
Materials, Device Architectures, Fabrication Methods, and Applications. Adv. Funct. Mater..

[8] Friedman H. C., Cosco E. D., Atallah T. L., Jia S., Sletten E. M., Caram J. R. (2021). Establishing Design Principles for Emissive Organic
SWIR Chromophores from Energy Gap Laws. Chem..

[9] Siddik A. B., Song W., Georgitzikis E., Vildanova M., Jin M., Berghmans F., Lieberman I., Malinowski P. E., Conard T., Cheyns D., Heremans P. (2025). InAs Colloidal Quantum
Dot Photodiode Stack for CMOS-Integrated Infrared Imaging. ACS Nano.

[10] Pradhan S., Dalmases M., Taghipour N., Kundu B., Konstantatos G. (2022). Colloidal
Quantum Dot Light Emitting Diodes at Telecom Wavelength with 18% Quantum
Efficiency and Over 1 MHz Bandwidth. Adv. Sci..

[11] Pradhan S., Di Stasio F., Bi Y., Gupta S., Christodoulou S., Stavrinadis A., Konstantatos G. (2019). High-Efficiency Colloidal Quantum
Dot Infrared Light-Emitting Diodes via Engineering at the Supra-Nanocrystalline
Level. Nat. Nanotechnol..

[12] Roshan H., Prudnikau A., Dai J., Cirignano M., De Boni F., Prato M., Paulus F., Lesnyak V., Di Stasio F. (2025). Short-Wave Infrared Optoelectronics
with Colloidal
CdHgSe/ZnCdS Core/Shell Nanoplatelets. ACS Photonics.

[13] Prudnikau, A. ; Roshan, H. ; Paulus, F. ; Martín-García, B. ; Hübner, R. ; Bahmani Jalali, H. ; De Franco, M. ; Prato, M. ; Di Stasio, F. ; Lesnyak, V. Efficient Near-Infrared Light-Emitting Diodes Based on CdHgSe Nanoplatelets. Adv. Funct. Mater. 2024, 34 (8), 10.1002/adfm.202310067.

[14] Qu J., Rastogi P., Gréboval C., Lagarde D., Chu A., Dabard C., Khalili A., Cruguel H., Robert C., Xu X. Z., Ithurria S., Silly M. G., Ferré S., Marie X., Lhuillier E. (2020). Electroluminescence
from HgTe Nanocrystals
and Its Use for Active Imaging. Nano Lett..

[15] Roshan H., Sheikhi M. H., Mirzaei A., Kaewmaraya T., Hussain T., Brescia R. (2023). Broadband Photodetection Using Metal
Excess Silver Sulfide Nanocrystals. J. Alloys
Compd..

[16] Roshan H., Ravanan F., Sheikhi M. H., Mirzaei A. (2021). High-Detectivity near-Infrared
Photodetector Based on Ag2S Nanocrystals. J.
Alloys Compd..

[17] Wang Y., Peng L., Schreier J., Bi Y., Black A., Malla A., Goossens S., Konstantatos G. (2024). Silver Telluride
Colloidal Quantum Dot Infrared Photodetectors and Image Sensors. Nat. Photonics.

[18] Paul, S. J. ; Li, L. ; Li, Z. ; Kywe, T. ; Vataj, A. ; Sahu, A. Heavy Metal Free Ag_2_ Se Quantum Dot Inks for Near to Short-Wave Infrared Detection. ACS Appl. Mater. Interfaces 2025, 17 (38), 53735 10.1021/acsami.5c12011. 40934373 PMC12464908

[19] Ma, Z. ; Sun, Z. ; Yang, H. ; Wang, Z. ; Ren, F. ; Yin, N. ; Chen, Q. ; Zhang, Y. ; Li, C. ; Chen, L. ; Wang, Q. Interface-Mediation-Enabled High-Performance Near-Infrared AgAuSe Quantum Dot Light-Emitting Diodes. J. Am. Chem. Soc. 2023, 145 (45), 10.1021/jacs.3c10214. 37910121

[20] Liu Z., Hao C., Sun Y., Wang J., Dube L., Chen M., Dang W., Hu J., Li X., Chen O. (2024). Rigid CuInS_2_ /ZnS Core/Shell
Quantum Dots for High Performance Infrared
Light-Emitting Diodes. Nano Lett..

[21] Bora A., Fu N., Saha A., Prudnikau A., Hübner R., Bahmani Jalali H., Di Stasio F., Gaponik N., Lesnyak V. (2025). Triangular-Shaped
Cu–Zn–In–Se-Based Nanocrystals with Narrow near
Infrared Photoluminescence. Nanoscale.

[22] Saha A., Cervino M., Roshan H., Hübner R., Wrzesińska-Lashkova A., Steinbach C., Vaynzof Y., Di Stasio F., Lesnyak V. (2025). RoHS-Compliant Cu-Zn-In-Se-Based
Core/Multi-shell Quantum Dots with Efficient and Tunable Short-Wave
Infrared Emission. Adv. Funct. Mater..

[23] Lim L. J., Zhao X., Tan Z. (2023). Non-Toxic
CuInS_2_ /ZnS
Colloidal Quantum Dots for Near-Infrared Light-Emitting Diodes. Adv. Mater..

[24] Kim T., Shin D., Kim M., Kim H., Cho E., Choi M., Kim J., Jang E., Jeong S. (2023). Development
of Group III–V Colloidal Quantum Dots for Optoelectronic Applications. ACS Energy Lett..

[25] Milnes A. G., Polyakov A. Y. (1993). Indium Arsenide:
A Semiconductor for High Speed and
Electro-Optical Devices. Mater. Sci. Eng., B.

[26] Kim T.-G., Zherebetskyy D., Bekenstein Y., Oh M. H., Wang L.-W., Jang E., Alivisatos A. P. (2018). Trap Passivation in Indium-Based
Quantum Dots through Surface Fluorination: Mechanism and Applications. ACS Nano.

[27] Jung D., Yu L., Wasserman D., Larry Lee M. (2015). Mid-Infrared Electroluminescence
from InAs Type-I Quantum Wells Grown on InAsP/InP Metamorphic Buffers. J. Appl. Phys..

[28] Krier A. (2001). Physics and
Technology of Mid–Infrared Light Emitting Diodes. Philos. Trans. R. Soc. London Ser. Math. Phys. Eng. Sci..

[29] Zhao X., Lim L. J., Ang S. S., Tan Z. (2022). Efficient Short-Wave
Infrared Light-Emitting Diodes Based on Heavy-Metal-Free Quantum Dots. Adv. Mater..

[30] Tessler N., Medvedev V., Kazes M., Kan S., Banin U. (2002). Efficient
Near-Infrared Polymer Nanocrystal Light-Emitting Diodes. Science.

[31] Battaglia D., Peng X. (2002). Formation of High Quality
InP and InAs Nanocrystals in a Noncoordinating
Solvent. Nano Lett..

[32] Zimmer J. P., Kim S.-W., Ohnishi S., Tanaka E., Frangioni J. V., Bawendi M. G. (2006). Size Series of Small
Indium Arsenide–Zinc Selenide
Core–Shell Nanocrystals and Their Application to In Vivo Imaging. J. Am. Chem. Soc..

[33] Panda, S. ; Sinatra, L. ; Yorov, K. E. ; Suwito, G. R. ; Bessonov, A. ; Lutfullin, M. ; Goldoni, L. ; Bergamaschi, E. ; Brescia, R. ; Prato, M. ; Divitini, G. ; De Trizio, L. ; Manna, L. Trioctylamine in the Synthesis of Tris­(Trimethylsilyl)­Arsine-Based InAs Quantum Dots Prevents the Formation of Si-Based Byproducts. J. Am. Chem. Soc. 2025, 147 (44), 40389 10.1021/jacs.5c11775. 41118469 PMC12593344

[34] Wijaya H., Darwan D., Zhao X., Ong E. W. Y., Lim K. R. G., Wang T., Lim L. J., Khoo K. H., Tan Z. (2020). Efficient
Near-Infrared Light-Emitting Diodes Based on In­(Zn)­As–In­(Zn)­P–GaP–ZnS
Quantum Dots. Adv. Funct. Mater..

[35] Li B., Wang Y., Zhang J., Li Y., Li B., Lin Q., Sun R., Fan F., Zeng Z., Shen H., Ji B. (2025). Efficient and Stable
Near-Infrared InAs Quantum Dot Light-Emitting
Diodes. Nat. Commun..

[36] Green M., Norager S., Moriarty P., Motevalli M., O’Brien P. (2000). On the Synthesis and Manipulation
of InAs Quantum Dots. J. Mater. Chem..

[37] Srivastava V., Dunietz E., Kamysbayev V., Anderson J. S., Talapin D. V. (2018). Monodisperse
InAs Quantum Dots from Aminoarsine Precursors: Understanding the Role
of Reducing Agent. Chem. Mater..

[38] Zhu D., Bellato F., Bahmani Jalali H., Di Stasio F., Prato M., Ivanov Y. P., Divitini G., Infante I., De Trizio L., Manna L. (2022). ZnCl_2_ Mediated Synthesis
of InAs Nanocrystals with Aminoarsine. J. Am.
Chem. Soc..

[39] De
Franco M., Zhu D., Asaithambi A., Prato M., Charalampous E., Christodoulou S., Kriegel I., De Trizio L., Manna L., Bahmani
Jalali H., Di Stasio F. (2022). Near-Infrared Light-Emitting Diodes
Based on RoHS-Compliant InAs/ZnSe Colloidal Quantum Dots. ACS Energy Lett..

[40] Zhu D., Bahmani Jalali H., Saleh G., Di Stasio F., Prato M., Polykarpou N., Othonos A., Christodoulou S., Ivanov Y. P., Divitini G., Infante I., De Trizio L., Manna L. (2023). Boosting the Photoluminescence
Efficiency of InAs Nanocrystals Synthesized
with Aminoarsine via a ZnSe Thick-Shell Overgrowth. Adv. Mater..

[41] Roshan H., Zhu D., Piccinotti D., Dai J., De Franco M., Barelli M., Prato M., De Trizio L., Manna L., Di Stasio F. (2024). Near Infrared Light-Emitting Diodes
Based on Colloidal InAs/ZnSe Core/Thick-Shell Quantum Dots. Adv. Sci..

[42] Skorotetcky M. S., Mir W. J., Sheikh T., Yorov K. E., Saidzhonov B. M., Daws S., Zhou R., Hedhili M. N., Abulikemu M., Mohammed O. F., Bakr O. M. (2025). Si-H Hydrosilane
Reducing Agents
for Size- and Shape-Controlled InAs Colloidal Quantum Dots. Adv. Mater..

[43] Kim T., Park S., Jeong S. (2021). Diffusion Dynamics Controlled Colloidal
Synthesis of Highly Monodisperse InAs Nanocrystals. Nat. Commun..

[44] Kim M., Lee J., Jung J., Shin D., Kim J., Cho E., Xing Y., Jeong H., Park S., Oh S. H., Kim Y.-H., Jeong S. (2024). Surface-Originated Weak Confinement
in Tetrahedral Indium Arsenide Quantum Dots. J. Am. Chem. Soc..

[45] Leemans J., Respekta D., Bai J., Braeuer S., Vanhaecke F., Hens Z. (2023). Formation of Colloidal
In­(As,P) Quantum Dots Active in the Short-Wave
Infrared, Promoting Growth through Temperature Ramps. ACS Nano.

[46] Panda S., Zhu D., Goldoni L., Asaithambi A., Brescia R., Saleh G., De Trizio L., Manna L. (2025). Overcoming the Short-Wave Infrared
Barrier in the Photoluminescence of Amino-As-Based InAs Quantum Dots. Adv. Opt. Mater..

[47] Shen W., Wang Y., Liao L. (2025). Near-Infrared
Quantum Dots for Electroluminescence:
Balancing Performance and Sustainability. Laser
Photonics Rev..

[49] Cunningham P. D., Coropceanu I., Mulloy K., Cho W., Talapin D. V. (2020). Quantized
Reaction Pathways for Solution Synthesis of Colloidal ZnSe Nanostructures:
A Connection between Clusters, Nanowires, and Two-Dimensional Nanoplatelets. ACS Nano.

[50] Schulz P., Tiepelt J. O., Christians J. A., Levine I., Edri E., Sanehira E. M., Hodes G., Cahen D., Kahn A. (2016). High-Work-Function
Molybdenum Oxide Hole Extraction Contacts in Hybrid Organic–Inorganic
Perovskite Solar Cells. ACS Appl. Mater. Interfaces.

[51] Olson D. C., Shaheen S. E., White M. S., Mitchell W. J., van Hest M. F. A. M., Collins R. T., Ginley D. S. (2007). Band-Offset Engineering for Enhanced
Open-Circuit Voltage in Polymer–Oxide Hybrid Solar Cells. Adv. Funct. Mater..

[52] Yarar Z. (2011). Steady-State
Electron Transport and Low-Field Mobility of Wurtzite Bulk ZnO and
Zn1–x Mg x O. J. Electron. Mater..

[53] Kılınç N., Arda L., Öztürk S., Öztürk Z. Z. (2010). Structure
and Electrical Properties of Mg-doped ZnO Nanoparticles. Cryst. Res. Technol..

[54] Kim J.-H., Han C.-Y., Lee K.-H., An K.-S., Song W., Kim J., Oh M. S., Do Y. R., Yang H. (2015). Performance Improvement
of Quantum Dot-Light-Emitting Diodes Enabled by an Alloyed ZnMgO Nanoparticle
Electron Transport Layer. Chem. Mater..

[55] Wu H., Zhang Y., Zhang X., Lu M., Sun C., Zhang T., Yu W. W. (2017). Enhanced Stability
and Performance
in Perovskite Nanocrystal Light-Emitting Devices Using a ZnMgO Interfacial
Layer. Adv. Opt. Mater..

[56] Meng L., Yao E., Hong Z., Chen H., Sun P., Yang Z., Li G., Yang Y. (2017). Pure Formamidinium-Based Perovskite Light-Emitting
Diodes with High Efficiency and Low Driving Voltage. Adv. Mater..

[57] Neumann K., Thelakkat M. (2014). Perovskite Solar Cells Involving Poly­(Tetraphenylbenzidine)­s:
Investigation of Hole Carrier Mobility, Doping Effects and Photovoltaic
Properties. RSC Adv..

[58] Tameev A. R., Aleksandrov A. E., Sayarov I. R., Pozin S. I., Lypenko D. A., Dmitriev A. V., Nekrasova N. V., Chernyadyev A. Yu., Tsivadze A. Yu. (2024). Charge Carrier
Mobility in Poly­(N,N′-Bis-4-Butylphenyl-N,N′-Bisphenyl)­Benzidine
Composites with Electron Acceptor Molecules. Polymers.

[59] Thesen M. W., Höfer B., Debeaux M., Janietz S., Wedel A., Köhler A., Johannes H., Krueger H. (2010). Hole-transporting Host-polymer
Series Consisting of Triphenylamine Basic Structures for Phosphorescent
Polymer Light-emitting Diodes. J. Polym. Sci.
Part Polym. Chem..

[60] Ryu S., Noh J. H., Jeon N. J., Chan Kim Y., Yang W. S., Seo J., Seok S. I. (2014). Voltage
Output of Efficient Perovskite Solar Cells
with High Open-Circuit Voltage and Fill Factor. Energy Env. Sci..

[61] Endres J., Kulbak M., Zhao L., Rand B. P., Cahen D., Hodes G., Kahn A. (2017). Electronic
Structure of the CsPbBr3/Polytriarylamine
(PTAA) System. J. Appl. Phys..

[62] Zhang W., Smith J., Hamilton R., Heeney M., Kirkpatrick J., Song K., Watkins S. E., Anthopoulos T., McCulloch I. (2009). Systematic Improvement in Charge
Carrier Mobility of
Air Stable Triarylamine Copolymers. J. Am. Chem.
Soc..

[63] Kuan C.-H., Luo G.-S., Narra S., Maity S., Hiramatsu H., Tsai Y.-W., Lin J.-M., Hou C.-H., Shyue J.-J., Wei-Guang Diau E. (2022). How Can a Hydrophobic Polymer PTAA Serve as a Hole-
Transport Layer for an Inverted Tin Perovskite Solar Cell?. Chem. Eng. J..

[64] Barard S., Heeney M., Chen L., Cölle M., Shkunov M., McCulloch I., Stingelin N., Philips M., Kreouzis T. (2009). Separate Charge Transport Pathways
Determined by the Time of Flight Method in Bimodal Polytriarylamine. J. Appl. Phys..

[65] Yuan C., Chen Z., Tian F., Chen S. (2024). Very Stable and Efficient
Tandem Quantum-Dot Light-Emitting Diodes Enabled by IZO-Based Interconnecting
Layers. Nano Lett..

[66] Shirasaki Y., Supran G. J., Bawendi M. G., Bulović V. (2013). Emergence
of Colloidal Quantum-Dot Light-Emitting Technologies. Nat. Photonics.

[67] Su Q., Zhang H., Chen S. (2025). Carrier Dynamics
in Quantum Dot Light-Emitting
Diodes: The Conversion between Electrons, Excitons, and Photons. Adv. Phys. Res..

