# Supplementary material for: Short-Wave Infrared InAs Quantum-Dot Light-Emitting Diodes with Tunable Electroluminescence beyond 1.4 μm
Source: ACS Energy Lett. 2026 Feb 4;11(5):3764–70. doi: 10.1021/acsenergylett.5c03820 (PMC13162317; doi:10.1021/acsenergylett.5c03820)
Supplement: Supplementary file 1 [file nz5c03820_si_001.pdf]

Supporting Information

Short-Wave Infrared InAs Quantum-Dot Light-Emitting Diodes with Tunable Electroluminescence Beyond 1.4 µm

Hossein Roshan[1], Davide Mazza[1,2], Satyaprakash Panda[2,3], Francesco de Boni[4], Luca De Trizio[5], Liberato Manna[3], Francesco Di Stasio[1]*

1. Photonic Nanomaterials, Istituto Italiano di Tecnologia, Via Morego 30, 16163 Genova, Italy

2. Università degli Studi di Genova, Via Dodecaneso 31, 16146 Genova, Italy

3. Nanochemistry, Istituto Italiano di Tecnologia, Via Morego 30, 16163 Genova, Italy

4. Materials Characterization Facility, Istituto Italiano di Tecnologia, Via Morego 30, Genova 16163, Italy

5. Chemistry Facility, Istituto Italiano di Tecnologia, Via Morego 30, 16163 Genova, Italy

*Corresponding Author's E-mail: francesco.distasio@iit.it

Experimental section:

Chemicals: Indium(III) chloride ($InCl_3$, 99.999%, Sigma-Aldrich/Merck), zinc(II) chloride ($ZnCl_2$, 99.999%, Sigma-Aldrich/Merck), zinc acetate dihydrate ($Zn(CH_3COO)_2{\cdot}2H_2O$, 99.99%, Sigma-Aldrich), magnesium acetate tetrahydrate ($Mg(CH_3COO)_2{\cdot}4H_2O$, 99.99%, Sigma-Aldrich), tetramethylammonium hydroxide (TMAH, Sigma-Aldrich), ethanolamine (≥99%, Sigma-Aldrich), dimethyl sulfoxide (DMSO, ≥99.9%, Sigma-Aldrich), tetrachloroethylene (anhydrous, >99%, Sigma-Aldrich), ethanol (anhydrous, ≥99.8%, Sigma-Aldrich), ethyl acetate (≥99.5%, Sigma-Aldrich), toluene (anhydrous, 99.8%, Sigma-Aldrich), chlorobenzene (anhydrous, 99.8%, Sigma-Aldrich), 1-octadecene (ODE, 90%, Sigma-Aldrich), tri-n-octylphosphine (TOP, 97%, Strem), trioctylamine (TOA, 98%, Sigma-Aldrich), aluminum(III) chloride ($AlCl_3$, 99.999%, Sigma-Aldrich), lithium aluminum hydride ($LiAlH_4$, 95%, Sigma-Aldrich), selenium powder (99.99%, Strem), oleylamine (OLA, 98%, Sigma-Aldrich) were used as received unless otherwise noted. The quantum-dot synthesis employed tris(dimethylamino)arsine (amino-As, 99%, Strem). Poly(methyl methacrylate) (PMMA, Sigma-Aldrich), poly(4-butyl-N,N-diphenylaniline) (Poly-TPD, Ossila), poly[bis(4-phenyl)(2,4,6-trimethylphenyl)amine] (PTAA, Ossila), molybdenum oxide (MoO_3, Alpha Chemicals), and chlorobenzene (anhydrous, 99.8%, Sigma-Aldrich) were employed in the light-emitting diodes fabrication.

InAs/ZnSe QD synthesis. InAs/ZnSe core/shell QDs were synthesized following the procedure reported in our previous work.[1] Briefly, InAs cores were prepared using amino-As as the arsenic precursor and trioctylamine-alane (TOA-AlH_3) adduct as the reducing agent. The $InCl_3$ precursor (0.2 M) was obtained by dissolving $InCl_3$ in oleylamine (OLA) at 250°C, while the amino-As precursor (0.1–0.4 M) was prepared by dissolving amino-As in degassed OLA inside a nitrogen-filled glovebox. The TOA-AlH_3 adduct was synthesized from $AlCl_3$ and $LiAlH_4$ in pentane, followed by the addition of trioctylamine and overnight stirring, as previously described.[1]

For the synthesis of the core, $InCl_3$ and $ZnCl_2$ precursors were mixed in OLA/ODE solvent mixture, degassed, and heated to 240°C under N_2. Amino-As was injected at this temperature, followed by the swift addition of TOA-AlH_3 in ODE. The reaction temperature was then raised to 280°C and maintained for 1 h. After completion, the reaction mixture was quenched to 90°C, and the desired amount of $ZnCl_2$-OLA and TOP-Se precursors were injected. The reaction was then reheated to 300°C and maintained for 2 h to grow the ZnSe shell. The resulting QDs (InAs/ZnSe QD1) were then cleaned using ethanol and toluene several times, before being stored in TCE under inert atmosphere.

Larger InAs QDs were obtained via a seeded growth approach by continuously injecting additional $InCl_3$-OLA, amino-As, and TOA-AlH_3 solutions at 240°C using syringe pumps to the above-synthesized core reaction mixture. The duration of the continuous injection varied depending on the desired QD size. After the growth, the resulting InAs cores were washed with toluene and ethanol and redispersed in OLA. The ZnSe shell was subsequently formed by injecting desired amounts of $ZnCl_2$-OLA and TOP-Se precursors and heating at 300°C for 2 hours. The resulting core/shell samples were purified as described above, yielding "InAs/ZnSe QD2", "InAs/ZnSe QD3", and "InAs/ZnSe QD4".

All the above steps were carried out under inert atmosphere using Schlenk-line or inside N_2-filled glovebox.

Optical Properties: Absorption spectra were collected with a Varian Cary 5000 UV-Vis-NIR spectrophotometer. For solution measurements, QD dispersions were prepared in 3 mL of tetrachloroethylene and transferred into 1 cm path-length quartz cuvettes with screw caps inside a N_2-filled glovebox. Solid-state samples were obtained by drop-casting the QD inks onto quartz substrates and subsequently encapsulating the films. PL was measured on an Edinburgh Instruments FLS900 spectrofluorometer equipped with a xenon lamp and an excitation monochromator. Absolute PL quantum yields (PLQYs) were determined on the same system using an integrating sphere with 700 nm excitation from a Xe lamp. For all solution-phase PL/PLQY measurements, dispersions were diluted to an optical density of ~0.1 at the excitation wavelength.

Electron-transport layer (ZnMgO): A clear solution was obtained by dissolving 2.70 mmol zinc acetate dihydrate (0.593 g) and 0.30 mmol magnesium acetate tetrahydrate (0.064 g) in 30 mL DMSO (total cations = 3.00 mmol; Zn:Mg = 0.9:0.1). A base mixture of 5 mL TMAH and 10 mL ethanol was prepared. Under vigorous stirring at room temperature, the TMAH/ethanol mixture was added slowly to the cation solution, and stirring was maintained for 1 h, upon which a colloidal $Zn_{0.9}Mg_{0.1}O$ dispersion was formed. The nanoparticles were precipitated by adding excess ethyl acetate, collected by centrifugation, the supernatant was discarded, and the solids were fully redispersed in ethanol. The ethanol dispersion was stored for subsequent characterization or film deposition.

Device fabrication: Optimized LEDs were fabricated on pre-patterned ITO substrates. The substrates were sequentially cleaned (laboratory detergent, acetone, and isopropanol), dried under nitrogen, and exposed to oxygen plasma for 10 min. The ETL was deposited by spin-coating a $Zn_{0.9}Mg_{0.1}O$ nanoparticle dispersion and annealing at 180 °C for 10 min. In a nitrogen glovebox, the InAs/ZnSe core/shell QD layer was deposited by spin-coating and briefly annealed at 100 °C (3 min). The double-HTL was formed by using Poly-TPD (high solubility in chlorobenzene and low solubility in toluene) and PTAA (high solubility in toluene). Poly-TPD (12 mg mL^{-1} in chlorobenzene) was spin-coated and annealed at 100 °C (3 min), followed by PTAA (8 mg mL^{-1} in toluene) spin-coating and a 75 °C annealing (5 min). Top contacts were deposited by thermal evaporation of 15 nm MoO_3 and 80 nm Au through a shadow mask. Devices were encapsulated by depositing 50 nm Al_2O_3 by atomic layer deposition (ALD).

Photoelectron spectroscopy and optical bandgaps: XPS measurements were carried out through a Kratos Axis UltraDLD spectrometer (Kratos Analytical Ltd.) with a monochromated Al Kα X-ray source (hν = 1486.6 eV) operating at 20 mA and 15 kV. Specimens were prepared by spin-coating QD solutions on a silicon substrate.

The wide scans were collected over an analysis area of 300 × 700 μm^2 at a photoelectron pass energy of 160 eV and an energy step of 1 eV, while high-resolution spectra were collected at a photoelectron pass energy of 10 eV and an energy step of 0.1 eV. A take-off angle of 0° with respect to sample normal direction was used for all analyses. The slight differential electrical charging effects (less than 0.5 eV) observed on all samples were not neutralized. The spectra have been referenced to the C-C component of the adventitious carbon 1s

peak at 284.8 eV. The spectra were analyzed with the CasaXPS software (Casa Software Ltd., version 2.3.25),[2] and the residual background was eliminated by the Shirley method across the binding energy range of the peaks of interest.

UPS measurements were performed using a He I (hν = 21.22 eV) discharge lamp, fitted in the same chamber used for XPS analyses, on an area of 55 µm in diameter, at a pass energy of 10 eV and with a dwell time of 100 ms.

The energy levels within the equipment and UPS-based calculations are described as follows. The work function, ϕ, i.e. the position of the Fermi level versus the vacuum level, was determined for each sample from the position of the secondary electron cutoff in the UPS spectrum, using the following equation: $\phi = h\nu - E_0$, where hν is the source energy (21.22 eV for He I photons) and E_0 is the secondary electron cut off.

Instead, the position of the valence band maximum (VBM) versus the vacuum level, i.e. the ionization energy, E_{ion}, was determined from the width of the entire UPS spectrum, according to the following equation: $E_{ion} = h\nu - (E_0 - E_1) = h\nu - E_0 + E_1$, where E_1 is the position of the VBM with respect to the zero (Fermi) level.[3] The values E_0 and E_1 were determined in the UPS spectrum through the background functions "Edge Up" and "Edge Down", respectively, in the CasaXPS software. The error bar associated with this procedure was estimated to be equal to 0.1 eV.

Electron microscopy: Bright-field TEM images of QDs were acquired on a JEOL JEM-1400 Plus (LaB_6, 120 kV) equipped with a Gatan Orius 830 CCD (2048 × 2048). Samples were prepared by drop-casting dilute QD dispersions onto carbon-coated Cu grids (200 mesh). Cross-sectional imaging of device stacks was performed using a FEI Helios NanoLab 650 dual-beam workstation (Elstar FESEM column and 30 kV Ga FIB). For sample preparation, the stack was protected with a thin Pt layer deposited before focused ion beam milling (FIB) to preserve the interfaces during cross-sectioning.

Device characterization: Devices (active area ≈ 4.5 mm^2 defined by electrode overlap) were biased with a Keithley 2636 source-measure unit while operated under ambient conditions (relative humidity 50%). Forward optical power was measured using a calibrated Ge photodiode (Gentec PH20-Ge-D0) positioned normal to the device surface. Assuming Lambertian emission, radiant intensity was derived from the measured forward power and collection geometry, and radiance was obtained by normalizing to the emissive area. EQE was calculated as the ratio of emitted photon flux to injected electron flux. Photon flux was computed from the forward light power, the peak EL wavelength, and Planck's relation under the same Lambertian assumption. Spectral measurements of EL were performed using an Edinburgh instrument FLS 900 equipped with a cooled-InGaAs-PMT.

Operational stability of LEDs: Stability tests were performed on encapsulated devices. Stability measurements were carried out in ambient air at ~50% relative humidity. All devices were driven under constant current operation at a fixed current density of 0.20 mA cm^{-2}, which corresponds to the peak EQE operating point for all devices. The EL intensity was recorded as a function of time and is reported as normalized intensity.

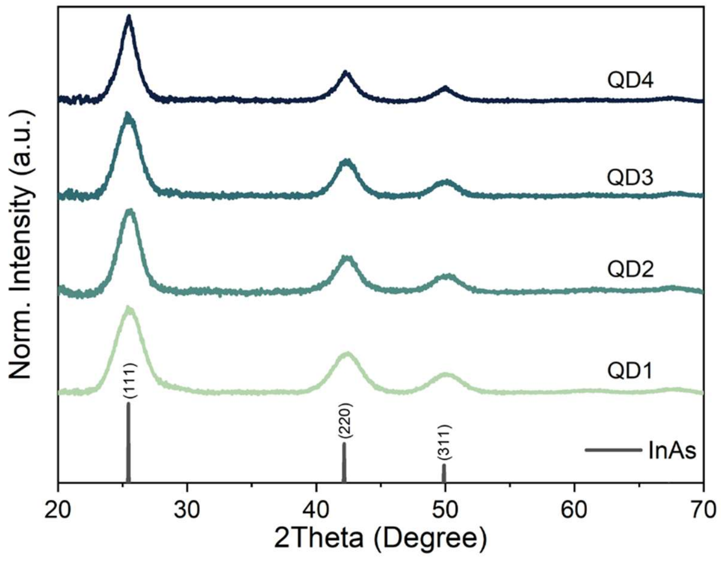

Figure S1. X-ray diffraction (XRD) patterns of the core InAs QDs.

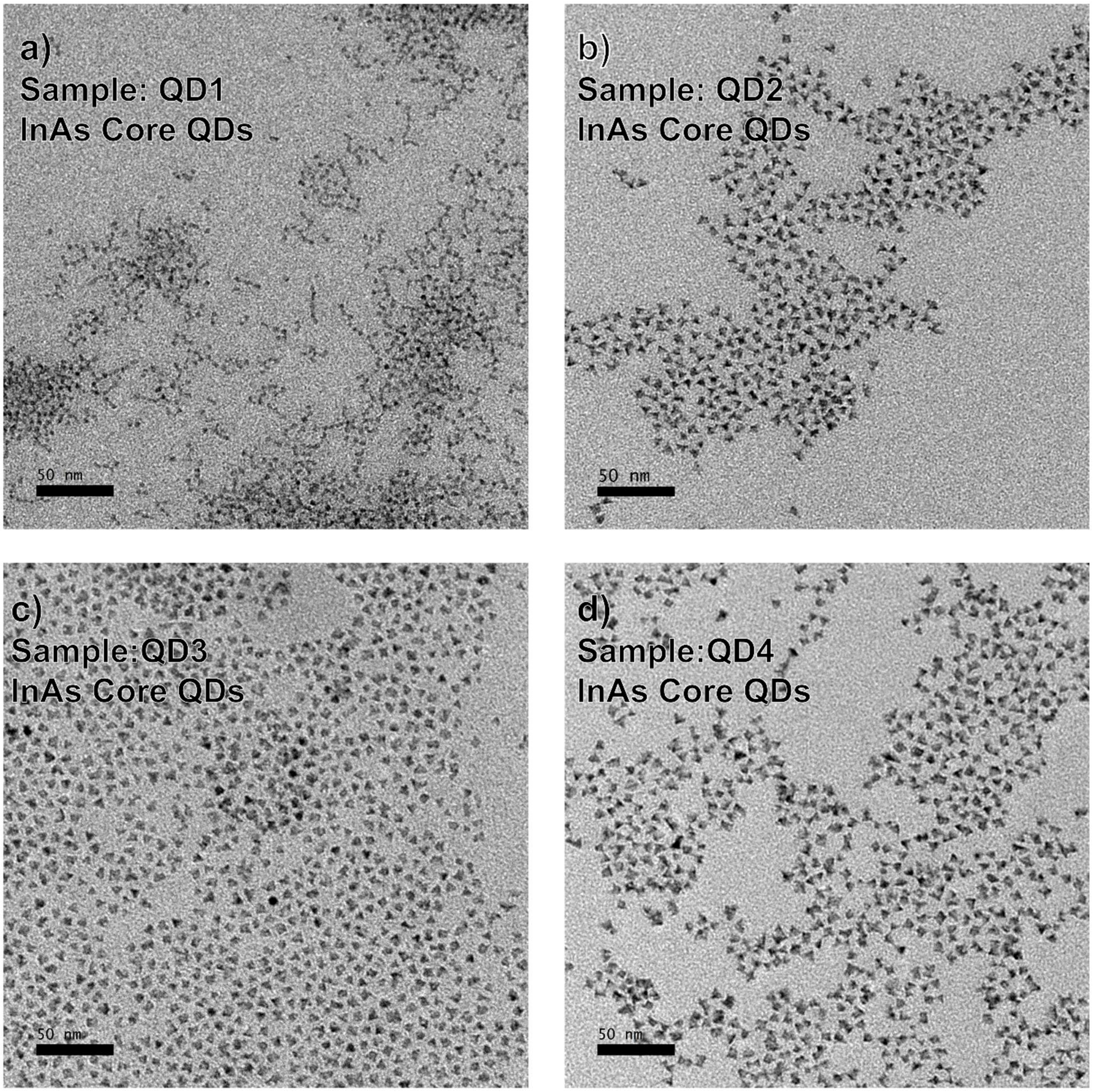

Figure S2. TEM image of core InAs QDs, with different sizes. All scale-bars are 50 nm.

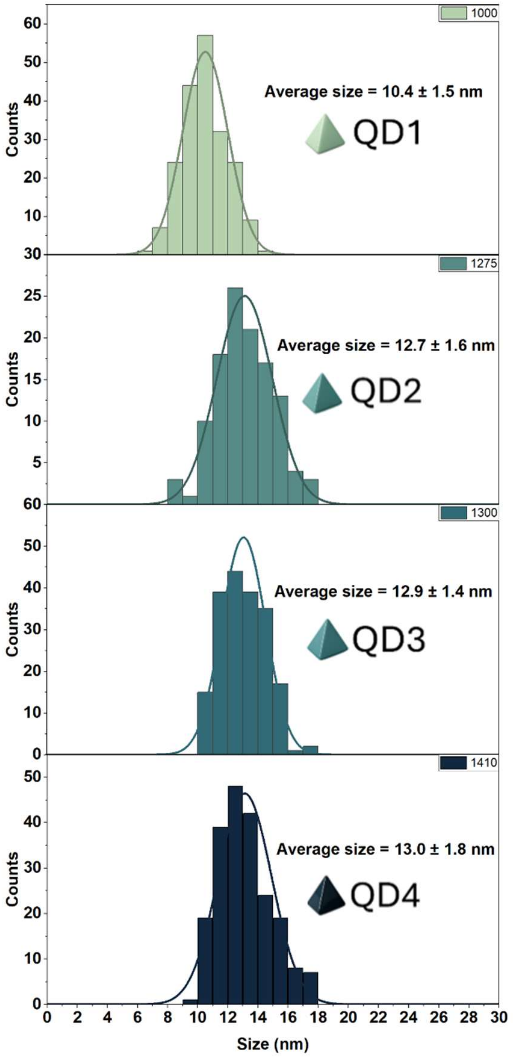

Figure S3. Size distribution histogram of core-shell QDs.

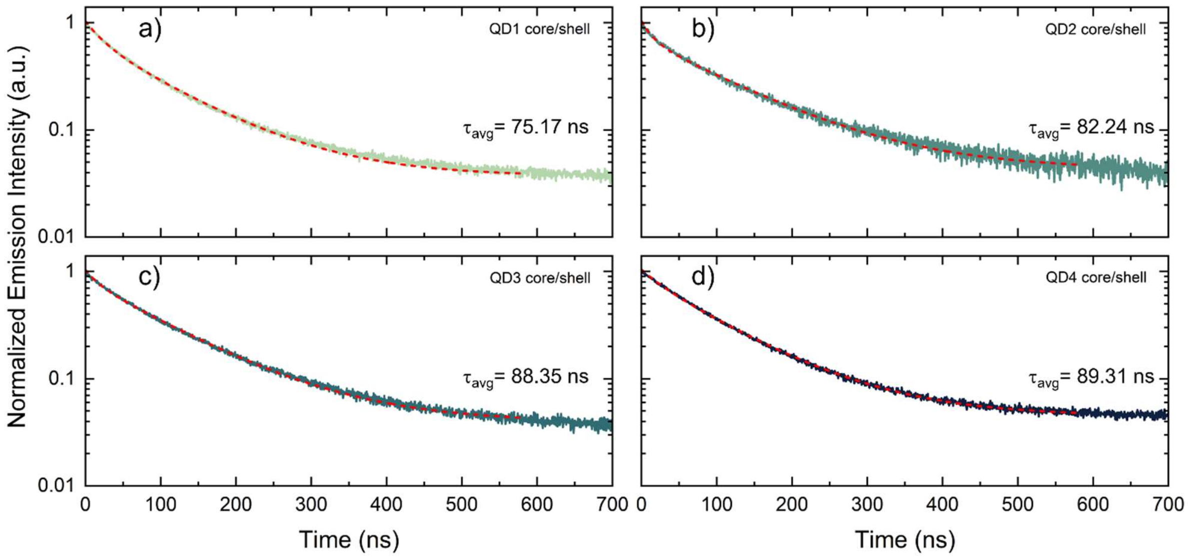

Figure S4. (a-d) Time resolved photoluminescence (TRPL) of InAs/ZnSe core/shell QDs (QD1-QD4) in solution (excitation wavelength = 505 nm). PL time-traces were fitted with an exponential decay function with 2 components: $y(t) = y_0 + A_1 e^{-\frac{t}{\tau_1}} + A_2 e^{-\frac{t}{\tau_2}}$. The two components were then used to calculate the amplitude average lifetime: $\tau_{avg} = \frac{A_1\tau_1 + A_2\tau_2}{A_1+A_2}$. A size-dependent trend was observed (in agreement with our previous publication)[1] with τ_{avg} increasing from around 75 ns to 89 ns from QD1 to QD4. The increase in photoluminescence lifetime is accompanied by a drop in PLQY, suggesting an increased content of shallow traps in large InAs/ZnSe core/shell QDs.

Table S1. Steady-state and time-resolved photoluminescence parameters of QD1-QD4.

Sample	PL peak (nm)	PLQY (%)	A_1	τ_1 (ns)	A_2	τ_2 (ns)	τ_{avg} (ns)
QD1	966	45 ± 5	0.33	21.81	0.67	101.47	75.17
QD2	1267	35 ± 4	0.33	13.53	0.67	116.10	82.24
QD3	1370	30 ± 3	0.22	15.41	0.78	108.93	88.35
QD4	1410	17 ± 2	0.18	25.85	0.82	103.25	89.31

XPS analysis:

XPS of core-only InAs QDs films (for QD1, QD2, QD3, and QD4) shows only In and As signals, (In $3d_{5/2,3/2}$ near at 444.5 - 452.1 eV, As $3d_{5/2,3/2}$ near 40–41 eV) with Zn 2p also detected, given its use during synthesis. After ZnSe shell growth, the spectra exhibit prominent Zn $2p_{3/2,1/2}$ at 1021.5 and 1044.6 eV and Se $3d_{5/2,3/2}$ at 54.5-55.4 eV, with attenuation of the In and As peaks, consistent with a ZnSe overlayer.

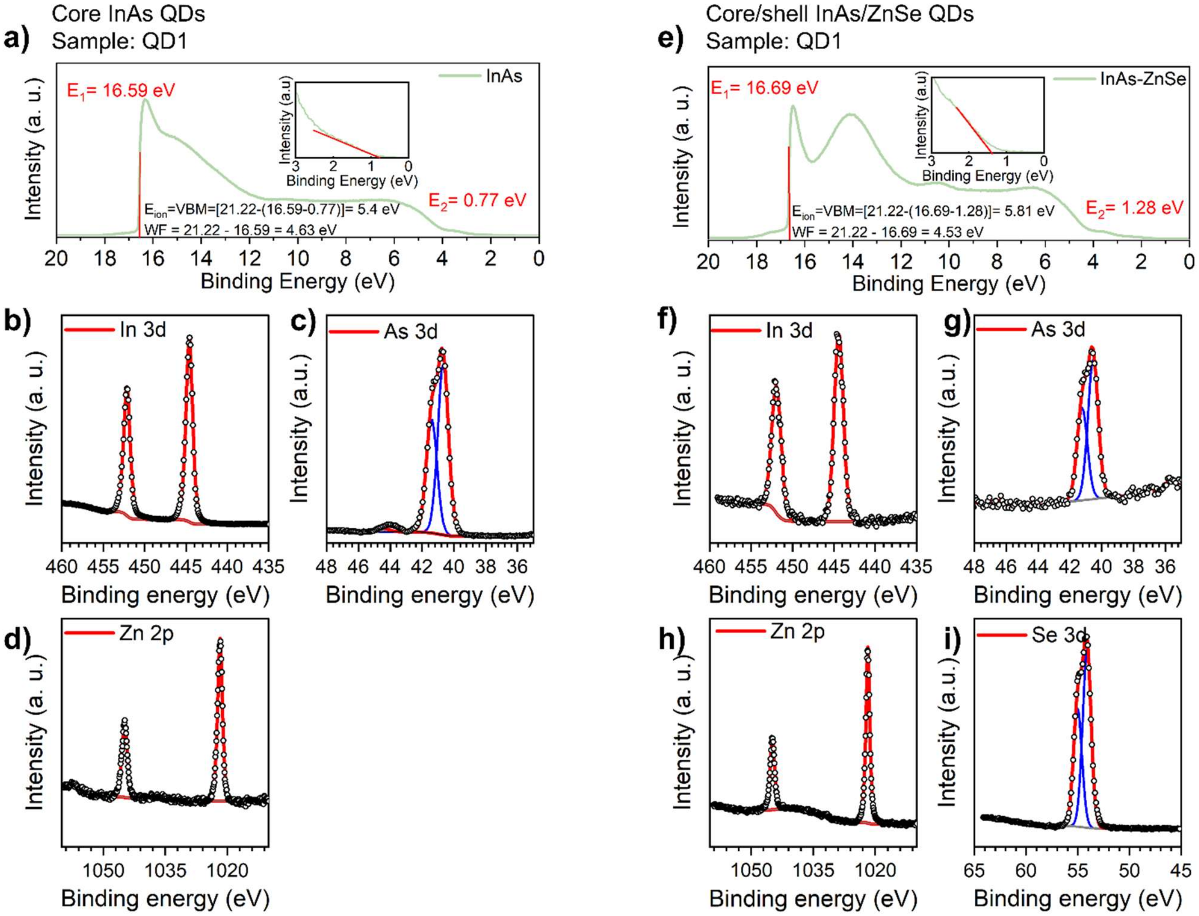

Figure S5. UPS/XPS of InAs cores vs InAs/ZnSe core/shell QDs QD1 sample. (a) UPS of InAs cores (He I, hv = 21.22 eV): secondary-electron cutoff and valence-band region were used to extract WF ≈ 4.6 eV and VBM ≈ 5.4 eV, according to the method explained in the Experimental Section of the main text. (b) In 3d doublet ($3d_{5/2}$, $3d_{3/2}$) of InAs cores. (c) As 3d doublet of InAs cores. (d) Zn 2p doublet of InAs cores, arising from surface Zn species from synthesis. (e) UPS of InAs/ZnSe core/shell QDs: WF ≈ 4.5 eV and VBM ≈ 5.8 eV. (f) In 3d doublet of core/shell QDs. (g) As 3d doublet of core/shell QDs. (h) Zn 2p doublet of core/shell QDs. (i) Se 3d doublet (~54-55 eV) confirming the ZnSe shell.

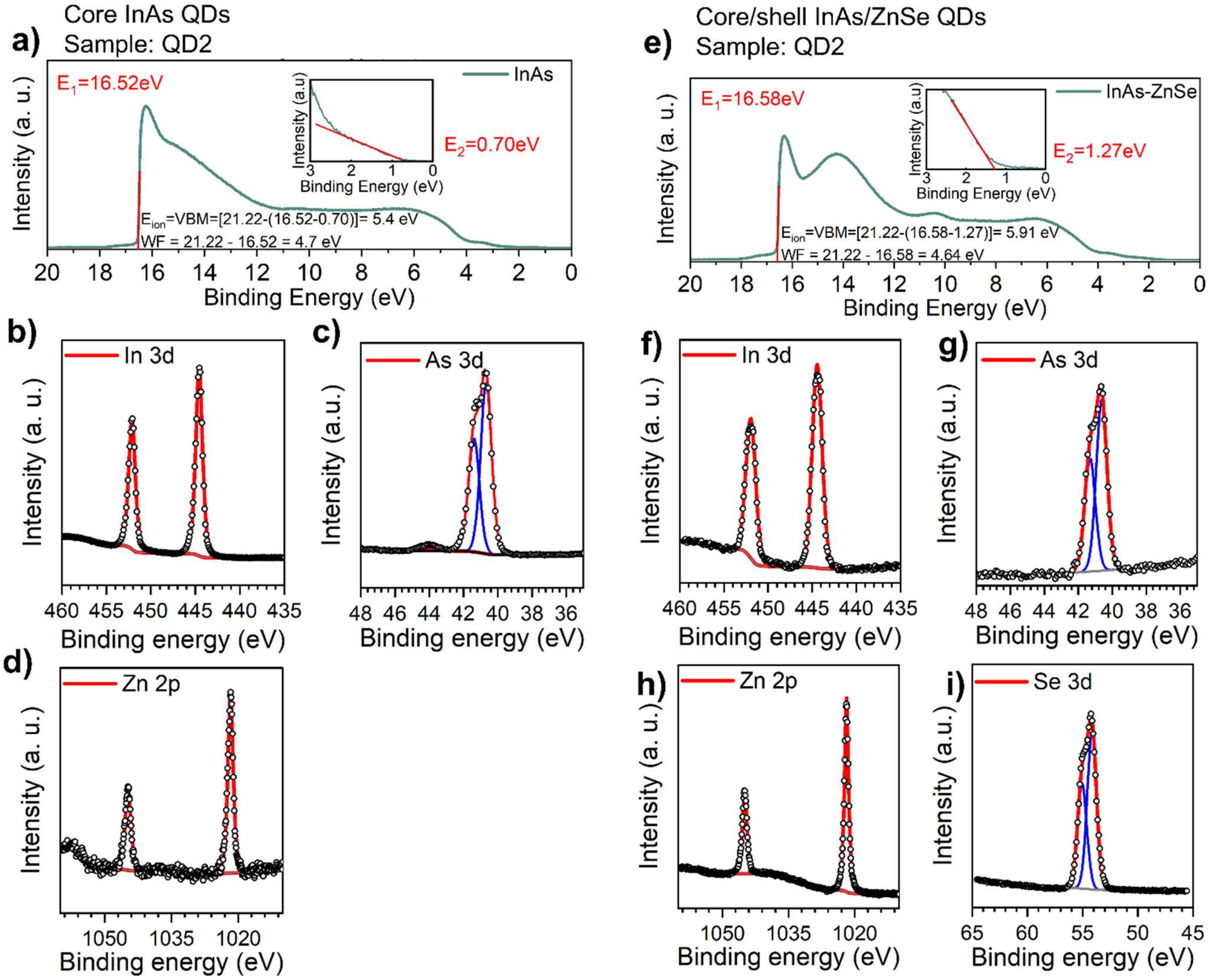

Figure S6. UPS/XPS of InAs cores vs InAs/ZnSe core/shell QDs QD2 sample. (a) UPS of InAs cores (He I, hν = 21.22 eV): secondary-electron cutoff and valence-band region were used to extract WF ≈ 4.7 eV and VBM ≈ 5.4 eV, according to the method explained in the Experimental Section of the main text. (b) In 3d doublet ($3d_{5/2}$, $3d_{3/2}$) of InAs cores. (c) As 3d doublet of InAs cores. (d) Zn 2p doublet of InAs cores, arising from surface Zn species from synthesis. (e) UPS of InAs/ZnSe core/shell QDs: WF ≈ 4.6 eV and VBM ≈ 5.9 eV. (f) In 3d doublet of core/shell QDs. (g) As 3d doublet of core/shell QDs. (h) Zn 2p doublet of core/shell QDs. (i) Se 3d doublet (~54-55 eV) confirming the ZnSe shell.

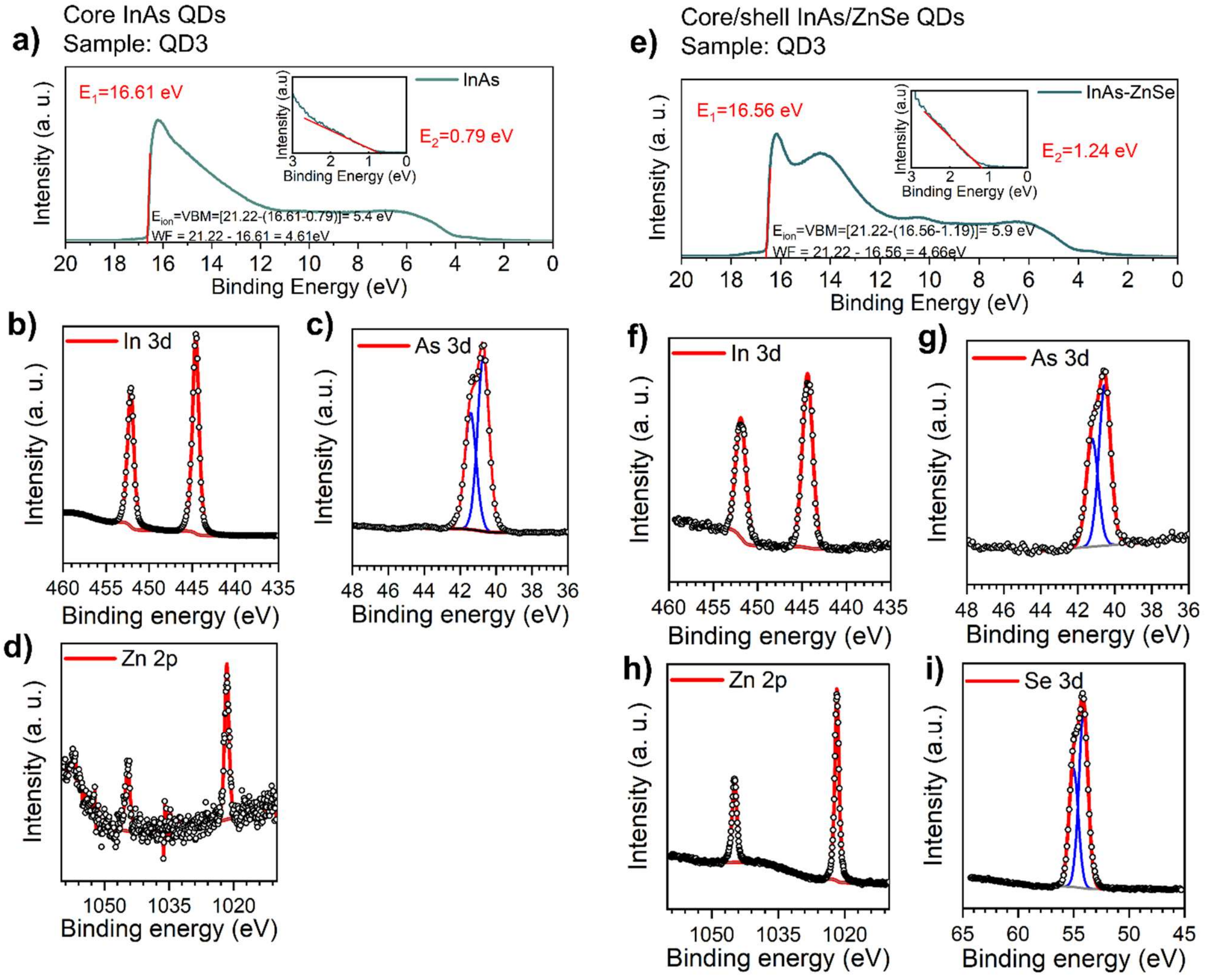

Figure S7. UPS/XPS of InAs cores vs InAs/ZnSe core/shell QDs QD3 sample. (a) UPS of InAs cores (He I, hν = 21.22 eV): secondary-electron cutoff and valence-band region were used to extract WF ≈ 4.6 eV and VBM ≈ 5.4 eV, according to the method explained in the Experimental Section of the main text. (b) In 3d doublet ($3d_{5/2}$, $3d_{3/2}$) of InAs cores. (c) As 3d doublet of InAs cores. (d) Zn 2p doublet of InAs cores, arising from surface Zn species from synthesis. (e) UPS of InAs/ZnSe core/shell QDs: WF ≈ 4.7 eV and VBM ≈ 5.9 eV. (f) In 3d doublet of core/shell QDs. (g) As 3d doublet of core/shell QDs. (h) Zn 2p doublet of core/shell QDs. (i) Se 3d doublet (~54-55 eV) confirming the ZnSe shell.

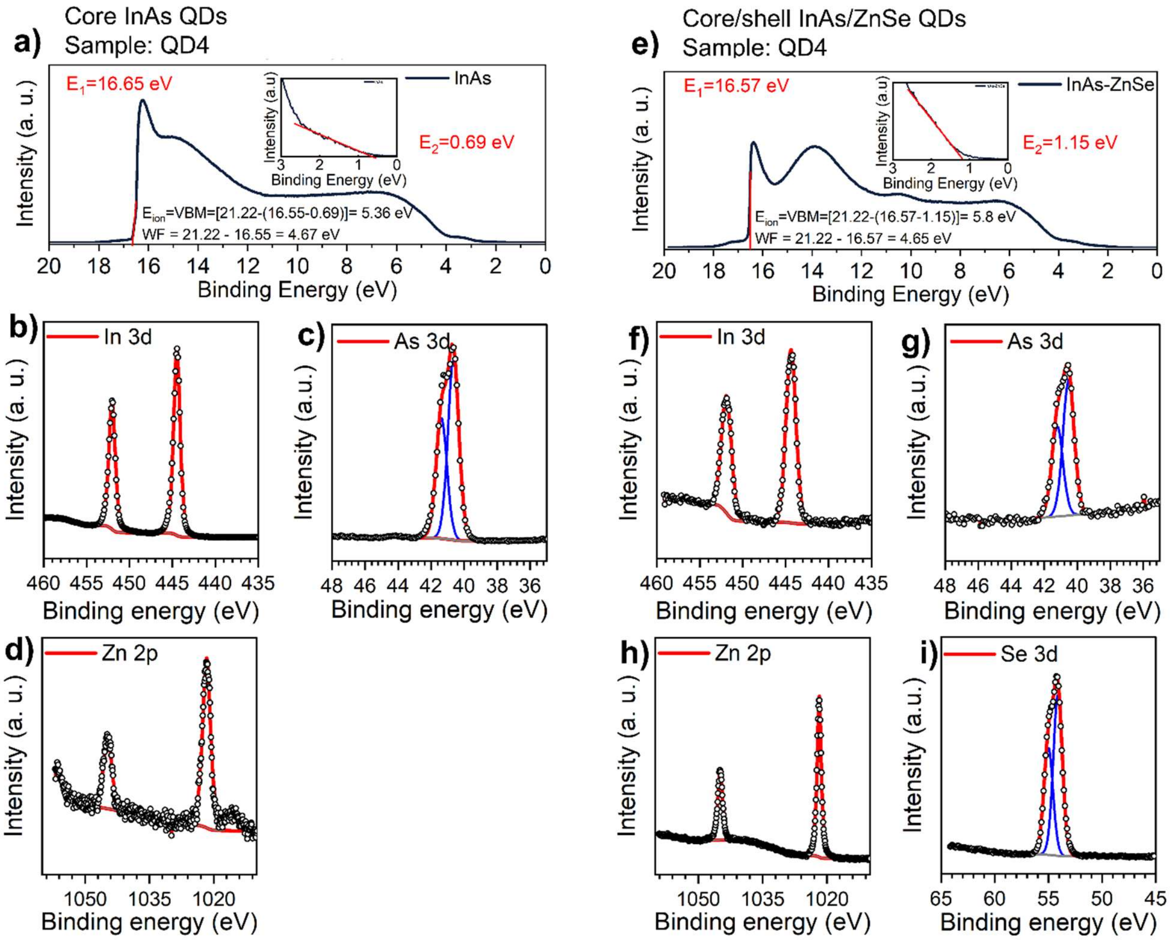

Figure S8. UPS/XPS of InAs cores vs InAs/ZnSe core/shell QDs QD4 sample. (a) UPS of InAs cores (He I, hν = 21.22 eV): secondary-electron cutoff and valence-band region were used to extract WF≈ 4.7 eV and VBM ≈ 5.4 eV, according to the method explained in the Experimental Section of the main text. (b) In 3d doublet ($3d_{5/2}$, $3d_{3/2}$) of InAs cores. (c) As 3d doublet of InAs cores. (d) Zn 2p doublet of InAs cores, arising from surface Zn species from synthesis. (e) UPS of InAs/ZnSe core/shell QDs: WF ≈ 4.7 eV and VBM ≈ 5.8 eV. (f) In 3d doublet of core/shell QDs. (g) As 3d doublet of core/shell QDs. (h) Zn 2p doublet of core/shell QDs. (i) Se 3d doublet (~54-55 eV) confirming the ZnSe shell.

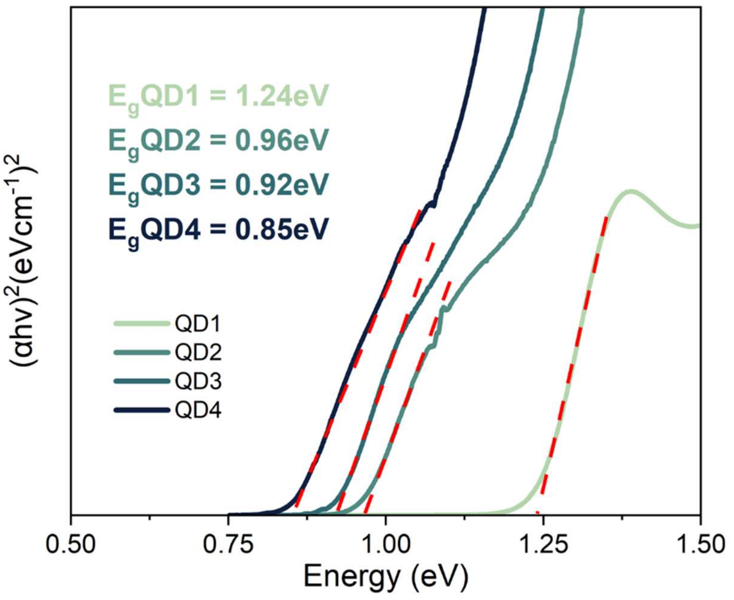

Figure S9. Tauc plot of core InAs QDs for determining the bandgap energy of QD1, QD2, QD3 and QD4.

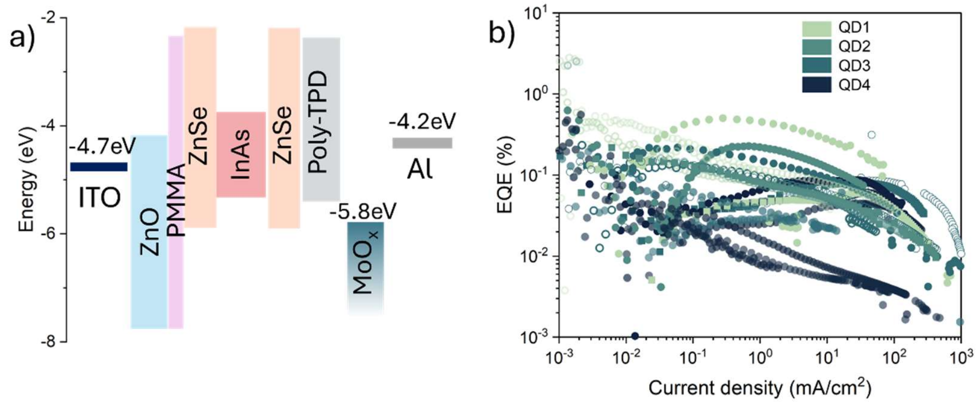

Figure S10. (a) Initial LED structure following our previous work [4], and (b) initial EQEs that were obtained from this architecture.

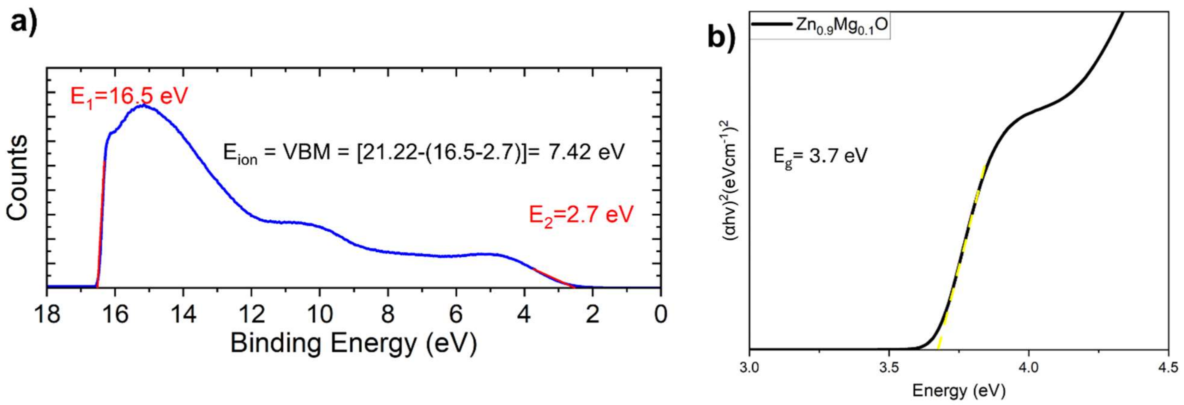

Figure S11. (a) UPS spectra of $Zn_{0.9}Mg_{0.1}O$ film with VBM of -7.4 eV. (b) Tauc plot and obtained bandgap energy of $Zn_{0.9}Mg_{0.1}O$ equal to 3.7 eV

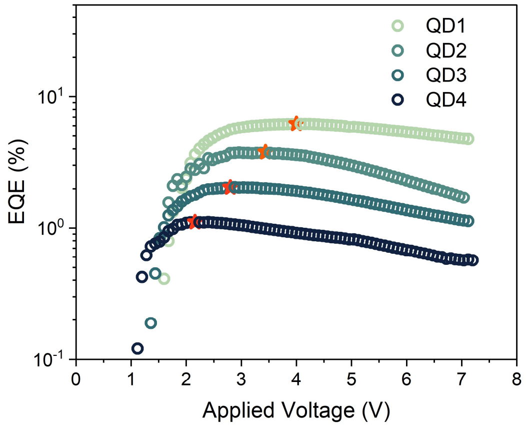

Figure S12. EQE versus applied bias. EQE peaks determined by orange stars.

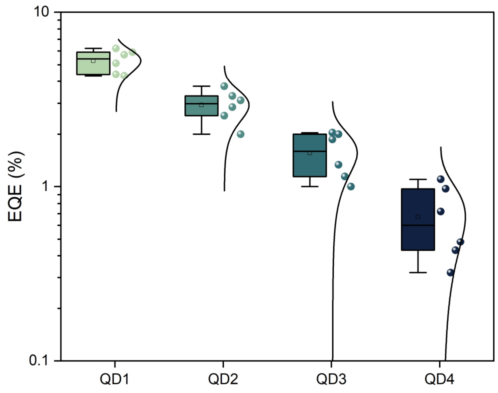

Figure S13. EQE statistic data of 6 pixels for each type of QD1, QD2, QD3, and QD4.

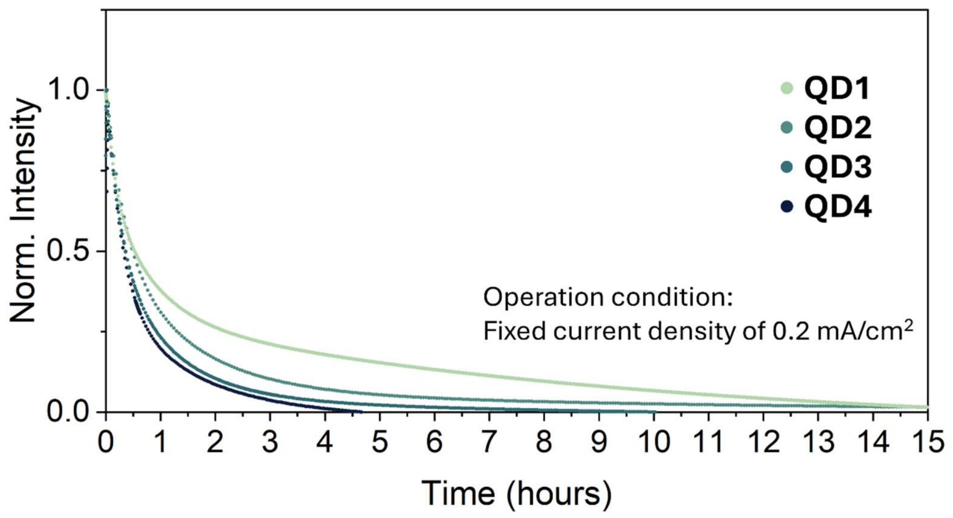

Figure S14. Operational stability test for each LED type: QD1, QD2, QD3, and QD4.

Table S2. Performance comparison among SWIR QD-LEDs.

Active QDs	EL peak (nm)	Max EQE (%)	RoHS-compliance	Ref.
HgTe	1000-1300	2.2	No (Hg)	[5]
HgTe	1400	0.25	No (Hg)	[6]
CdHgSe/ZnCdS	1300	7	No (Cd, Hg)	[7]
PbS-CdS	1200	4.3	No (Pb,Cd)	[8]
PbS	1400	7.9	No (Pb)	[9]
PbS	1550	11.8	No (Pb)	[10]
AgAuSe	1046	15.8	Yes	[11]
$CsSnI_3$	950	3.8	Yes	[12]
$CsSnI_3$+PEAI	920	3	Yes	[13]
$CsSnI_3$	931	5.6	Yes	[14]
$FASnI_3$	866	5.3	Yes	[15]
$CsSnI_3$	948	2.63	Yes	[16]
$FACsSnI_3$	894	8.3	Yes	[17]
$FACsSnI_3$	898	11.6	Yes	[18]
Tin-Halide Perovskites	909	12.4	Yes	[19]
$CuInS_2$/ZnS	920	8.2	Yes	[20]
$CuInS_2$/ZnS	940	2.6	Yes	[21]
InAs/ZnSe	1270	0.5	Yes	[22]
In(Zn)As/In(Zn)P/GaP/ZnS	850	4.6	Yes	[23]
InAs/ZnSe	947	5.5	Yes	[24]
In(Zn)As/In(Zn)P/GaP/ZnS	1006	13.3	Yes	[25]
InAs/ZnSe	900	13.3	Yes	[4]
InAs/InP/ZnSe/ZnS	900	20.5	Yes	[26]
**InAs/ZnSe**	**1007-1410**	**6.2 – 1.1**	**Yes**	**This work**

References

(1) Panda, S.; Zhu, D.; Goldoni, L.; Asaithambi, A.; Brescia, R.; Saleh, G.; De Trizio, L.; Manna, L. Overcoming the Short-Wave Infrared Barrier in the Photoluminescence of Amino-As-Based InAs Quantum Dots. *Advanced Optical Materials* **2025**, e01512. https://doi.org/10.1002/adom.202501512.

(2) Fairley, N.; Fernandez, V.; Richard-Plouet, M.; Guillot-Deudon, C.; Walton, J.; Smith, E.; Flahaut, D.; Greiner, M.; Biesinger, M.; Tougaard, S.; Morgan, D.; Baltrusaitis, J. Systematic and Collaborative Approach to Problem Solving Using X-Ray Photoelectron Spectroscopy. *Applied Surface Science Advances* **2021**, *5*, 100112. https://doi.org/10.1016/j.apsadv.2021.100112.

(3) Dandrade, B.; Datta, S.; Forrest, S.; Djurovich, P.; Polikarpov, E.; Thompson, M. Relationship between the Ionization and Oxidation Potentials of Molecular Organic Semiconductors. *Organic Electronics* **2005**, *6* (1), 11–20. https://doi.org/10.1016/j.orgel.2005.01.002.

(4) Roshan, H.; Zhu, D.; Piccinotti, D.; Dai, J.; De Franco, M.; Barelli, M.; Prato, M.; De Trizio, L.; Manna, L.; Di Stasio, F. Near Infrared Light-Emitting Diodes Based on Colloidal InAs/ZnSe Core/Thick-Shell Quantum Dots. *Advanced Science* **2024**, *11* (23), 2400734. https://doi.org/10.1002/advs.202400734.

(5) Bossavit, E.; Qu, J.; Abadie, C.; Dabard, C.; Dang, T.; Izquierdo, E.; Khalili, A.; Gréboval, C.; Chu, A.; Pierini, S.; Cavallo, M.; Prado, Y.; Parahyba, V.; Xu, X. Z.; Decamps-Mandine, A.; Silly, M.; Ithurria, S.; Lhuillier, E. Optimized Infrared LED and Its Use in an All-HgTe Nanocrystal-Based Active Imaging Setup. *Advanced Optical Materials* **2022**, *10* (4), 2101755. https://doi.org/10.1002/adom.202101755.

(6) Prado, Y.; Qu, J.; Gréboval, C.; Dabard, C.; Rastogi, P.; Chu, A.; Khalili, A.; Xu, X. Z.; Delerue, C.; Ithurria, S.; Lhuillier, E. Seeded Growth of HgTe Nanocrystals for Shape Control and Their Use in Narrow Infrared Electroluminescence. *Chem. Mater.* **2021**, *33* (6), 2054–2061. https://doi.org/10.1021/acs.chemmater.0c04526.

(7) Roshan, H.; Prudnikau, A.; Dai, J.; Cirignano, M.; De Boni, F.; Prato, M.; Paulus, F.; Lesnyak, V.; Di Stasio, F. Short-Wave Infrared Optoelectronics with Colloidal CdHgSe/ZnCdS Core/Shell Nanoplatelets. *ACS Photonics* **2025**, *12* (1), 40–47. https://doi.org/10.1021/acsphotonics.4c01944.

(8) Supran, G. J.; Song, K. W.; Hwang, G. W.; Correa, R. E.; Scherer, J.; Dauler, E. A.; Shirasaki, Y.; Bawendi, M. G.; Bulović, V. High-Performance Shortwave-Infrared Light-Emitting Devices Using Core–Shell (PbS–CdS) Colloidal Quantum Dots. *Advanced Materials* **2015**, *27* (8), 1437–1442. https://doi.org/10.1002/adma.201404636.

(9) Pradhan, S.; Di Stasio, F.; Bi, Y.; Gupta, S.; Christodoulou, S.; Stavrinadis, A.; Konstantatos, G. High-Efficiency Colloidal Quantum Dot Infrared Light-Emitting Diodes via Engineering at the Supra-Nanocrystalline Level. *Nature Nanotech* **2019**, *14* (1), 72–79. https://doi.org/10.1038/s41565-018-0312-y.

(10) Pradhan, S.; Dalmases, M.; Taghipour, N.; Kundu, B.; Konstantatos, G. Colloidal Quantum Dot Light Emitting Diodes at Telecom Wavelength with 18% Quantum Efficiency and Over 1 MHz Bandwidth. *Advanced Science* **2022**, *9* (20), 2200637. https://doi.org/10.1002/advs.202200637.

(11) Ma, Z.; Sun, Z.; Yang, H.; Wang, Z.; Ren, F.; Yin, N.; Chen, Q.; Zhang, Y.; Li, C.; Chen, L.; Wang, Q. Interface-Mediation-Enabled High-Performance Near-Infrared AgAuSe Quantum Dot Light-Emitting Diodes. *J. Am. Chem. Soc.* **2023**, jacs.3c10214. https://doi.org/10.1021/jacs.3c10214.

(12) Hong, W.; Huang, Y.; Chang, C.; Zhang, Z.; Tsai, H.; Chang, N.; Chao, Y. Efficient Low-Temperature Solution-Processed Lead-Free Perovskite Infrared Light-Emitting Diodes. *Advanced Materials* **2016**, *28* (36), 8029–8036. https://doi.org/10.1002/adma.201601024.

(13) Wang, Y.; Zou, R.; Chang, J.; Fu, Z.; Cao, Y.; Zhang, L.; Wei, Y.; Kong, D.; Zou, W.; Wen, K.; Fan, N.; Wang, N.; Huang, W.; Wang, J. Tin-Based Multiple Quantum Well Perovskites for Light-Emitting Diodes with Improved Stability. *J. Phys. Chem. Lett.* **2019**, *10* (3), 453–459. https://doi.org/10.1021/acs.jpclett.8b03700.

(14) Li, Y.; Guan, X.; Meng, Y.; Chen, J.; Lin, J.; Chen, X.; Liu, C.; Zhao, Y.; Zhang, Q.; Tian, C.; Lu, J.; Wei, Z. Boosting $CsSnI_3$ -based Near-infrared Perovskite Light-emitting Diodes Performance via Solvent Coordination Engineering. *InfoMat* **2024**, *6* (5), e12537. https://doi.org/10.1002/inf2.12537.

(15) Zhang, F.; Min, H.; Zhang, Y.; Kuang, Z.; Wang, J.; Feng, Z.; Wen, K.; Xu, L.; Yang, C.; Shi, H.; Zhuo, C.; Wang, N.; Chang, J.; Huang, W.; Wang, J. Vapor-Assisted In Situ Recrystallization for Efficient Tin-Based

Perovskite Light-Emitting Diodes. *Advanced Materials* **2022**, *34* (37), 2203180. https://doi.org/10.1002/adma.202203180.
(16) Yuan, F.; Folpini, G.; Liu, T.; Singh, U.; Treglia, A.; Lim, J. W. M.; Klarbring, J.; Simak, S. I.; Abrikosov, I. A.; Sum, T. C.; Petrozza, A.; Gao, F. Bright and Stable Near-Infrared Lead-Free Perovskite Light-Emitting Diodes. *Nat. Photon.* **2024**, *18* (2), 170–176. https://doi.org/10.1038/s41566-023-01351-5.
(17) Min, H.; Chang, J.; Tong, Y.; Wang, J.; Zhang, F.; Feng, Z.; Bi, X.; Chen, N.; Kuang, Z.; Wang, S.; Yuan, L.; Shi, H.; Zhao, N.; Qian, D.; Xu, S.; Zhu, L.; Wang, N.; Huang, W.; Wang, J. Additive Treatment Yields High-Performance Lead-Free Perovskite Light-Emitting Diodes. *Nat. Photon.* **2023**, *17* (9), 755–760. https://doi.org/10.1038/s41566-023-01231-y.
(18) Min, H.; Wang, N.; Chen, N.; Tong, Y.; Wang, Y.; Wang, J.; Liu, J.; Wang, S.; Wu, X.; Yang, P.; Shi, H.; Zhuo, C.; Chen, Q.; Li, J.; Zhang, D.; Lu, X.; Zhu, C.; Peng, Q.; Zhu, L.; Chang, J.; Huang, W.; Wang, J. Spin Coating Epitaxial Heterodimensional Tin Perovskites for Light-Emitting Diodes. *Nat. Nanotechnol.* **2024**, *19* (5), 632–637. https://doi.org/10.1038/s41565-023-01588-9.
(19) Wang, H.; Treglia, A.; Wu, C.-S. J.; Zheng, G.; De Vries Ibáñez, M. M.; Vilé, G.; Li, H.; Gregori, L.; De Angelis, F.; Wang, J.; Gao, F.; Petrozza, A. In-Situ Self-Encapsulated Tin-Halide Perovskites for Air-Functional Near-Infrared Light-Emitting Diodes. *ACS Energy Lett.* **2025**, *10* (7), 3375–3382. https://doi.org/10.1021/acsenergylett.5c01017.
(20) Lim, L. J.; Zhao, X.; Tan, Z. Non-Toxic $CuInS_2$ /ZnS Colloidal Quantum Dots for Near-Infrared Light-Emitting Diodes. *Advanced Materials* **2023**, *35* (28), 2301887. https://doi.org/10.1002/adma.202301887.
(21) Liu, Z.; Hao, C.; Sun, Y.; Wang, J.; Dube, L.; Chen, M.; Dang, W.; Hu, J.; Li, X.; Chen, O. Rigid $CuInS_2$ /ZnS Core/Shell Quantum Dots for High Performance Infrared Light-Emitting Diodes. *Nano Lett.* **2024**, *24* (17), 5342–5350. https://doi.org/10.1021/acs.nanolett.4c01249.
(22) Tessler, N.; Medvedev, V.; Kazes, M.; Kan, S.; Banin, U. Efficient Near-Infrared Polymer Nanocrystal Light-Emitting Diodes. *Science* **2002**, *295* (5559), 1506–1508. https://doi.org/10.1126/science.1068153.
(23) Wijaya, H.; Darwan, D.; Zhao, X.; Ong, E. W. Y.; Lim, K. R. G.; Wang, T.; Lim, L. J.; Khoo, K. H.; Tan, Z. Efficient Near-Infrared Light-Emitting Diodes Based on In(Zn)As–In(Zn)P–GaP–ZnS Quantum Dots. *Adv Funct Materials* **2020**, *30* (4), 1906483. https://doi.org/10.1002/adfm.201906483.
(24) De Franco, M.; Zhu, D.; Asaithambi, A.; Prato, M.; Charalampous, E.; Christodoulou, S.; Kriegel, I.; De Trizio, L.; Manna, L.; Bahmani Jalali, H.; Di Stasio, F. Near-Infrared Light-Emitting Diodes Based on RoHS-Compliant InAs/ZnSe Colloidal Quantum Dots. *ACS Energy Lett.* **2022**, *7* (11), 3788–3790. https://doi.org/10.1021/acsenergylett.2c02070.
(25) Zhao, X.; Lim, L. J.; Ang, S. S.; Tan, Z. Efficient Short-Wave Infrared Light-Emitting Diodes Based on Heavy-Metal-Free Quantum Dots. *Advanced Materials* **2022**, *34* (45), 2206409. https://doi.org/10.1002/adma.202206409.
(26) Li, B.; Wang, Y.; Zhang, J.; Li, Y.; Li, B.; Lin, Q.; Sun, R.; Fan, F.; Zeng, Z.; Shen, H.; Ji, B. Efficient and Stable Near-Infrared InAs Quantum Dot Light-Emitting Diodes. *Nat Commun* **2025**, *16* (1), 2450. https://doi.org/10.1038/s41467-025-57746-1.
